# SIRM/SIC consensus document on the management of patients with acute chest pain

**DOI:** 10.1007/s11547-025-02076-x

**Published:** 2025-09-23

**Authors:** Antonio Esposito, Riccardo Faletti, Anna Palmisano, Marco Gatti, Sara Seitun, Cesare Mantini, Piergiuseppe Agostoni, Daniele Andreini, Francesco Barillà, Andrea Barison, Paolo Calabrò, Matteo Cameli, Scipione Carerj, Carlo Catalano, Marcello Chiocchi, Marco Matteo Ciccone, Antonio Curcio, Fabrizio D’Ascenzo, Serena Dell’Aversana, Fabio Falzea, Marco Francone, Nicola Galea, Andrea Giovagnoni, Marco Guglielmo, Andrea Laghi, Carlo Liguori, Luigi Lovato, Riccardo Marano, Rocco Antonio Montone, Doralisa Morrone, Luigi Natale, Savina Nodari, Michele Oppizzi, Stefania Paolillo, Alberto Polimeni, Gianluca Pontone, Italo Porto, Silvia Pradella, Vincenzo Russo, Vincenzo Russo, Luca Saba, Gianfranco Sinagra, Massimo Slavich, Carmen Spaccarotella, Davide Tore, Davide Vignale, Carmine Dario Vizza, Saverio Muscoli, Pasquale Perrone Filardi, Ciro Indolfi

**Affiliations:** 1 Experimental Imaging Centre, Radiology Unit, IRCCS San Raffaele Scientific Institute, Vita-Salute San Raffaele University, Milan, Italy; 2 Department of Surgical Sciences, Radiology Unit, University of Turin, Via Genova 3, 10126 Turin, Italy; 3 Department of Radiology, IRCCS Ospedale Policlinico San Martino, Genoa, Italy; 4 Department of Neuroscience, Imaging and Clinical Sciences, ‘G. d’Annunzio’ University, Chieti, Italy; 5 Monzino Cardiology Center, IRCCS, Milan, Italy; 6 Division of University Cardiology, IRCCS Ospedale Galeazzi Sant’Ambrogio, Milan, Italy; 7 Department of Systems Medicine, University of Rome Tor Vergata, Rome, Italy; 8 Cardiology and Cardiovascular Medicine Department, Fondazione Toscana Gabriele Monasterio, Pisa, Italy; 9 Interdisciplinary Center for Health Science, Scuola Superiore Sant’Anna, Pisa, Italy; 10 Sant’Anna E San Sebastiano Hospital, Caserta, Italy; 11 Division of Cardiology, University of Campania “Luigi Vanvitelli”, Caserta, Italy; 12 Department of Medical Biotechnologies, Division of Cardiology, University of Siena, Siena, Italy; 13 Department of Clinical and Experimental Medicine, University of Messina, Messina, Italy; 14 Department of Radiological, Oncological and Pathological Sciences, “La Sapienza” University of Rome, Rome, Italy; 15 Department of Biomedicine and Prevention, Division of Diagnostic Imaging, University of Rome “Tor Vergata”, Rome, Italy; 16 Cardiology Unit, Department of Precision and Regenerative Medicine and Ionian Area (DiMePRe-J), University of Bari Medical School, Bari, Italy; 17 Division of Cardiology, Department of Pharmacy, Health and Nutritional Sciences, University of Calabria, 87036 Rende, Italy; 18 Division of Cardiology, Cardiovascular and Thoracic Department, “Città Della Salute E Della Scienza” Hospital, Turin, Italy; 19 Department of Medical Sciences, University of Turin, Turin, Italy; 20 Department of Radiology, Santa Maria Delle Grazie Hospital, ASL Napoli 2 Nord, Pozzuoli, Italy; 21 Centro ECORAD Di Villa San Giovanni, Reggio Calabria, Italy; 22 Department of Biomedical Sciences, Humanitas University, Pieve Emanuele, Italy; 23 Department of Radiology, University Hospital “Ospedali Riuniti”, Ancona, Italy; 24 Division of Heart and Lungs, Department of Cardiology, Utrecht University Medical Center, Utrecht, The Netherlands; 25 Department of Medical Surgical Sciences and Translational Medicine, Sapienza–University of Rome, Radiology Unit–Sant’Andrea University Hospital, Rome, Italy; 26 UOC Diagnostica Per Immagini, P.O. San Giovanni Bosco-ASL Na1 Centro, Napoli, Via F.M. Briganti 255, 80144 Naples, Italy; 27 Department of Pediatric and Adult Cardio-Thoracovascular, Oncohematologic and Emergencies Radiology Unit, IRCCS Azienda Ospedaliero–Universitaria di Bologna, Bologna, Italy; 28 Department of Radiological and Hematological Sciences, Section of Radiology, Università Cattolica del Sacro Cuore, Rome, Italy; 29 Department of Cardiovascular and Pulmonary Sciences, Catholic University of the Sacred Heart, Rome, Italy; 30 Department of Cardiovascular Sciences, Fondazione Policlinico Universitario A. Gemelli IRCCS, Rome, Italy; 31 Department of Surgical, Medical and Molecular Pathology and of Critical Sciences, University of Pisa, Pisa, Italy; 32 Department of Radiological Sciences, Institute of Radiology, Catholic University of Rome, A. Gemelli University Hospital, Rome, Italy; 33 Department of Cardiology, University of Brescia and ASST Spedali Civili Di Brescia, Brescia, Italy; 34 Unit of Clinical Cardiology, IRCCS San Raffaele, Milan, Italy; 35 Department of Advanced Biomedical Sciences, University of Naples Federico II, Naples, Italy; 36 Division of Interventional Cardiology, Annunziata Hospital, Cosenza, Italy; 37 Department of Perioperative Cardiology and Cardiovascular Imaging, Centro Cardiologico Monzino IRCSS, Milan, Italy; 38 Cardiovascular Disease Unit, IRCCS Ospedale Policlinico San Martino, IRCCS Italian Cardiology Network, Genoa, Italy; 39 Department of Radiology, Careggi University Hospital, Florence, Italy; 40 Cardiology Unit, Department of Medical Translational Sciences, University of Campania “Luigi Vanvitelli”—Monaldi Hospital, Naples, Italy; 41 Department of Radiology, Azienda Ospedaliero Universitaria, University of Cagliari, Cagliari, Italy; 42 Cardiovascular Department, Azienda Sanitaria Universitaria Giuliano-Isontina, University of Trieste, Trieste, Italy; 43 Department of Cardiology, IRCCS San Raffaele Scientific Institute, Milan, Italy; 44 Radiology Unit, Department of Diagnostic Imaging and Interventional Radiology, “Città della Salute e della Scienza” - Molinette Hospital, Turin, Italy; 45 Department of Cardiovascular and Respiratory Sciences, Sapienza University of Rome, Rome, Italy; 46 Department of Cardiology, Policlinico Tor Vergata, Rome, Italy; 47 IRCCS Humanitas Research Hospital, Rozzano, Italy

**Keywords:** Acute chest pain, Acute coronary syndrome, Coronary Computed Tomography Angiography (CCTA), Cardiac Magnetic Resonance (CMR), Emergency department, Consensus statement

## Abstract

Acute chest pain is a common and challenging reason for emergency department visits and requires prompt and systematic evaluation to address potential life-threatening conditions, minimize risks and manage emergency department overcrowding. This updated consensus statement outlines the appropriate management of patients presenting to the emergency department with acute chest pain, emphasizing the timing and utility of non-invasive advanced imaging (particularly coronary computed tomography angiography) aiming to improve rapid and accurate diagnosis of both cardiac or non-cardiac causes improving patient safety, outcomes, and resource utilization efficiency. The writing committee was composed of members and experts from both the Italian Society of Cardiology (SIC) and the Italian Society of Medical and Interventional Radiology (SIRM) who worked jointly to create a cohesive approach in the field of acute chest pain. This structured approach may streamline diagnostic workflows in the emergency setting and support earlier, more appropriate patient management.

**Supplementary Information:**

The online version contains supplementary material available at 10.1007/s11547-025-02076-x.

## Introduction

Acute chest pain (ACP) is one of the most frequent causes of emergency department (ED) admissions and represents a clinically challenging condition. ACP can be the symptom of life-threatening pathophysiological processes requiring immediate intervention, as well as the expression of a minor clinical conditions, sometimes exacerbated by psychological factors such as stress or anxiety. Therefore, patients presenting with ACP should never be underestimated and must receive immediate attention. At the same time, a rational approach is essential to avoid unnecessary risks and prevent ED overcrowding, as widely documented, taking advantage of new technologies and modern clinical-diagnostic approaches to achieve maximum efficiency [1]. The need for a unified clinical-diagnostic pathway among specialists involved in managing this urgent condition has led to the creation of this document. Developed collaboratively by the Italian Society of Cardiology (SIC) and the Italian Society of Medical and Interventional Radiology (SIRM), this document focuses on defining diagnostic pathways, with particular emphasis on non-invasive imaging techniques.

The sections regarding the clinical evaluation of a patient with ACP and the first- and second-line diagnostic exams are available as supplementary data.

## Definition, epidemiology, and etiologies of ACP

ACP is defined as non-traumatic chest pain of new onset or acutely changed in its pattern of onset, intensity, or duration. The term “ACP” refers to a broad range of thoracic symptoms, including true precordial pain or sensations such as pressure, tightness, heaviness, crushing, or burning. These sensations may also occur outside the chest and radiate to other areas of the body, such as the shoulder, arm, neck, upper abdomen, or jaw. ACP represents approximately 8–10% of annual ED admissions among patients aged over 18 years old, with roughly 5% of these caused by acute coronary syndrome (ACS) [2]. Therefore, in most cases, ACP originates from non-cardiac causes, while about 15% of cases remain undiagnosed [3]. It has been estimated that more than one-fifth of medico-legal expenses are related to the inappropriate discharge of patients with ACS from EDs. Thus, an accurate assessment of ACP is essential to stratify the risk of ACS, a condition that can be life-threatening for the patient. While clinical presentation, electrocardiographic findings, and cardiac necrosis markers can sometimes provide diagnostic confirmation of acute myocardial infarction (AMI), they are sometimes insufficient to distinguish ACS from the many potential cardiac or non-cardiac etiologies of ACP.

## Preliminary assessment of patients with ACP

### History, pain characteristics, and clinical examination

The initial clinical evaluation of a patient with ACP should focus on the rapid identification and treatment of potentially life-threatening conditions such as ACS, aortic dissection (AD), pulmonary embolism (PE) and infarct-like myocarditis.

**Fig. 1:**
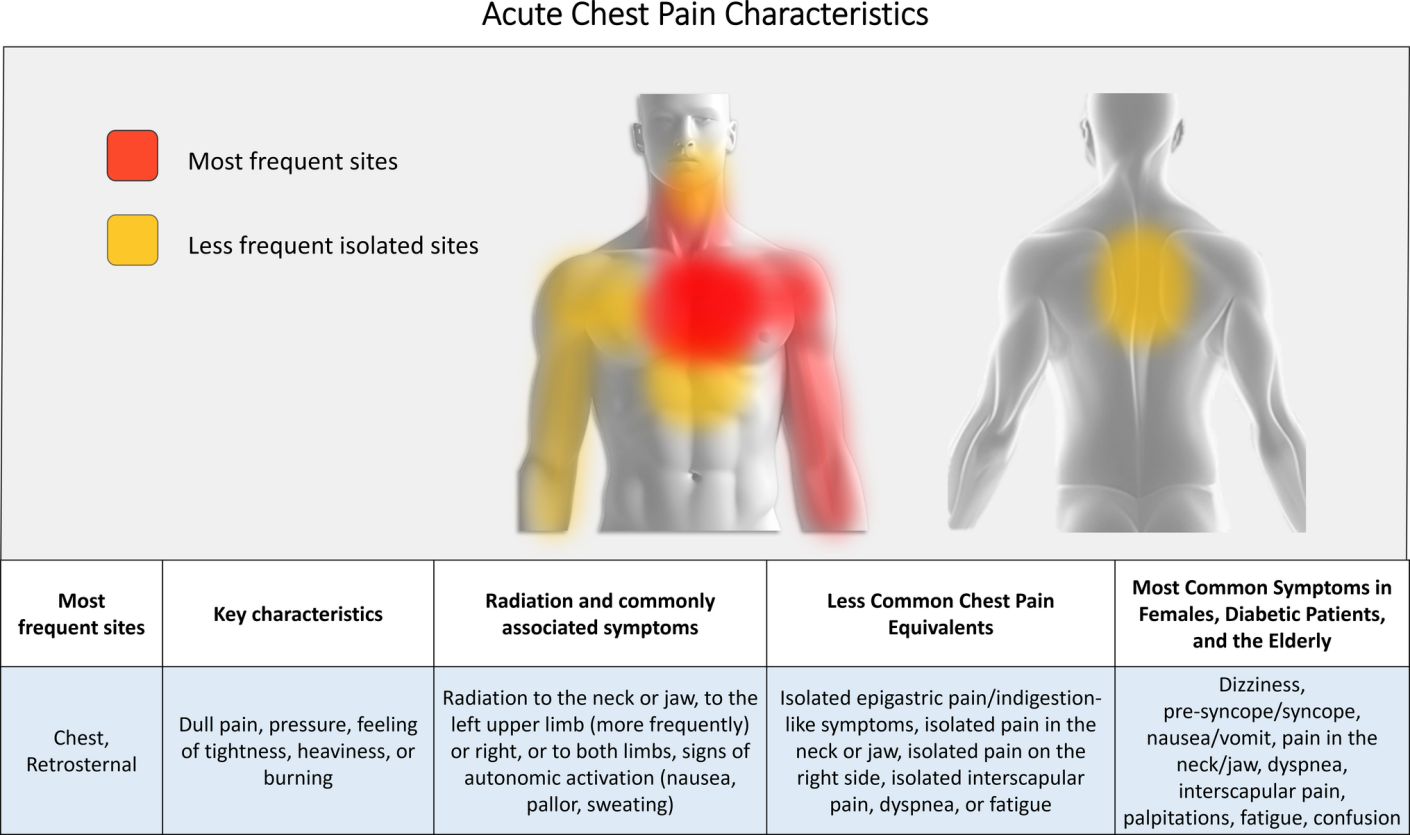
Acute Chest Pain Characteristics

Symptoms described as chest pressure, tightness, heaviness, or burning should be considered indicative of ACS (Fig. 1). In cases of ACS, the pain may also manifest in other areas of the body, such as the shoulder, arm, neck, back, upper abdomen, or jaw. Other potentially associated symptoms include dyspnea, nausea, vomiting, sweating, fatigue, or altered mental status, which can sometimes be the main presenting symptom. Conversely, symptoms described as hyperacute, fleeting, related to inspiration (pleuritic) or position, or localized to a single point are unlikely related to myocardial ischemia. Chest pain is traditionally classified as “typical” or “atypical.” However, according to the 2021 international guidelines from the AHA/ACC/ASE/CHEST/SAEM/SCCT/SCM [2], it is now preferable to use the terms “cardiac,” “possibly cardiac,” and “non-cardiac” to describe the likely cause of chest pain. Chest pain should be considered stable when symptoms are chronic and associated with identifiable triggers such as physical exertion or emotional stress. The clinical assessment should include a detailed description of chest pain and related symptoms, including their onset, duration, location, radiation, and alleviating or exacerbating factors. Additionally, a complete evaluation of cardiovascular risk factors and the patient’s medical history should supplement the symptom analysis. Any results from previous diagnostic tests for coronary artery disease (CAD) should also be reviewed.

The clinical assessment should aim to identify signs associated with a high-risk of morbidity and mortality, such as:

- signs of reduced cardiac output, (e.g., tachycardia, hypotension, cold extremities, low urine output, altered mental status)
- signs of heart failure (e.g., pulmonary edema, elevated jugular venous pressure, peripheral edema)
- new onset of a new systolic murmur suggestive of acute mitral regurgitation or a ventricular septal defect

Signs suggesting alternative etiologies to ACS include:

- fever (endocarditis or pneumonia)
- differential blood pressure between pulses (aortic dissection)
- pulmonary abnormalities detected on auscultation or chest X-ray (pneumonia or pneumothorax)
- pericardial friction rub (pericarditis)
- or other cardiac murmurs (e.g., aortic stenosis, endocarditis).

### Electrocardiogram (ECG)

Electrocardiogram (ECG) is a cornerstone of the initial assessment and management of patients presenting with ACP. For patients with suspected ACS, an ECG should be performed and interpreted as soon as possible [2].

The ECG must be promptly analyzed to identify signs of acute infarction or ischemia. If such signs are present, further management should proceed according to current guidelines for ST-elevation myocardial infarction (STEMI) or non-ST- elevation ACS (NSTE-ACS), which includes non-ST-elevation myocardial infarction (NSTEMI) and unstable angina (UA). Patients with non-ischemic ECG patterns should undergo a clinical decision pathway based on additional evaluations.

**Fig. 2:**
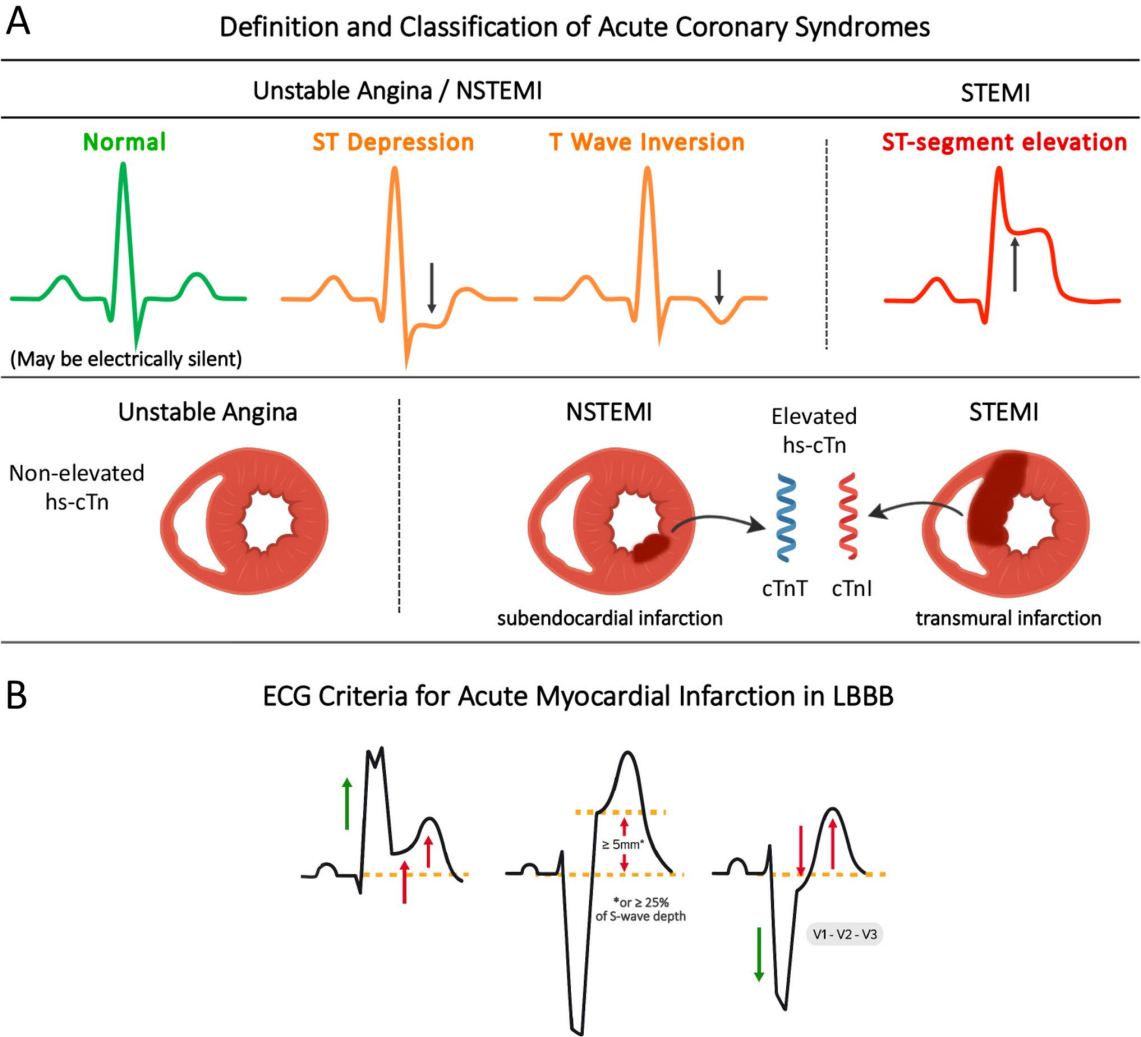
Classification and ECG Criteria in Patients with Acute Coronary Syndrome. Panel A. This panel illustrates the classification of acute coronary syndromes (ACS) based on ECG changes, cardiac biomarkers, and the extent of myocardial injury. Patients with unstable angina or NSTEMI may present with ST-segment depression or T-wave inversion on ECG, though the ECG may appear normal or with nonspecific ST-T changes. In NSTEMI, elevated high-sensitivity cardiac troponins (hs-cTnT or hs-cTnI) indicate subendocardial infarction, typically caused by partial coronary occlusion. STEMI is characterized by ST-segment elevation and transmural infarction due to complete coronary occlusion. Elevated hs-cTn refers to values above the 99th percentile of the upper reference limit. Panel B. ECG examples illustrating the original and Smith‑modified Sgarbossa criteria for AMI in left bundle branch block (LBBB) or ventricular‑paced rhythms. The left illustration shows concordant ST elevation ≥ 1 mm in leads with a positive QRS complex; the middle illustration shows excessively discordant ST elevation ≥ 5 mm (original criterion) or ≥ 25% of the depth of the preceding S‑wave (Smith‑modified criterion) in leads with a predominantly negative QRS complex; and the right illustration shows concordant ST depression ≥ 1 mm in leads V1–V3. *Abbreviations* AMI, acute myocardial infarction; ACS, acute coronary syndrome; ECG, electrocardiogram; hs-cTn, high-sensitivity cardiac troponin; LBBB, left bundle branch block; NSTEMI, non-ST-segment elevation myocardial infarction; STEMI, ST- segment elevation myocardial infarction

ECGs can be classified into three categories (Fig. 2):

1. STEMI or STEMI-equivalent
2. Ischemic ST-segment or T-wave abnormalities
3. Non-ischemic patterns, which include normal ECGs, non-specific findings, left ventricular hypertrophy (with or without ST-T segment changes), left or right bundle branch block, or ventricular paced rhythm that do not meet the Sgarbossa criteria [4] or modified Sgarbossa criteria [5] for AMI.

In the setting of UA/NSTEMI: (1) UA diagnosis is based on the presence of new, worsening, resting chest pain or occurring with minimal exertion without any elevation in biomarkers indicating myocardial injury or necrosis. (2) The presence of ECG abnormalities increases the diagnostic probability; however, ECG changes may be completely absent in more than one-third of patients with NSTEMI. (3) The prognostic value of T-wave inversions is lower compared to ST-segment depression. ST-segment depression also serves as a quantitative prognostic marker of risk (number of leads used and severity of ST-segment depression).

In patients with STEMI, the sum of ST-segment elevations across all leads (ΣSTE) is a clinical marker of ischemic myocardium and myocardium-at-risk.

### Troponins

High-sensitivity cardiac troponin T (hs-cTnT) and high-sensitivity cardiac troponin I (hs-cTnI) are the preferred serum biomarkers for evaluating patients with suspected ACS. Troponin concentrations should be reported as whole numbers in nanograms per liter (ng/L). Sex-specific 99th percentile cut-offs are recommended to increase diagnostic sensitivity in women and specificity in men. Serial measurement using 0h/1h or 0h/2h algorithms are recommended for timely rule-in and rule-out [6].

### 1st level imaging: chest X-ray and echocardiography

Both chest X-ray and echocardiography are low cost and widely available first level imaging modalities that provides very useful information in patients with ACP able to help the differential diagnosis, to detect signs with prognostic value and to guide the patient’s management. A description about the role of chest X-ray and echocardiography in the setting of ACP is reported in the supplementary materials (supplementary 3.4.1 and 3.4.2, respectively).

## Risk stratification using clinical and laboratory data-based algorithms

The pre-test probability assessment, combined with the calculation of risk scores, are critical elements in evaluating ACP patients. For instance, in the setting of suspected angina, the ESC guidelines recommend an assessment of pre-test probability of CAD, based on age, sex, and pain characteristics [7]. The kind of pain and any associated gesturing (Levine sign) can guide clinicians in formulating a diagnostic hypothesis. However, these are just probability estimates based on very simple criteria; therefore, even a high pre-test probability of CAD does not necessarily mean that CAD is the cause of the patient’s symptoms and ED access, and vice-versa in the case of low pre-test probability. The chest pain score aims to facilitate the differentiation between ischemic and non-ischemic chest pain [8]. A description of most used scores is reported in the supplementary materials (supplementary 4.1) and supplementary Table 1 (Table S1).

## 2nd level imaging: coronary CT angiography, cardiac magnetic resonance, stress imaging

Technological and methodological advances have made coronary CT angiography (CCTA) the clinical reference standard for ruling-out obstructive CAD. Hence, CCTA is considered a safe and effective diagnostic tool highly recommended in patients with low-to-intermediate risk ACP and normal troponins/ECG, allowing to reduce hospital stay and costs. Moreover, CCTA, eventually integrated with emerging advanced scanning techniques like late contrast enhancement CT and stress-CT perfusion, plays a growing role in managing “troponinosis,” preventing unnecessary catheterizations and improving differential diagnosis. This CCTA complementary role is particularly important in the current high-sensitivity troponins (hs-cTn) era, because the wide adoption of hs-cTn has improved sensitivity in the diagnosis of ACS, but also increases false positives, making CCTA a valuable complementary tool to reduce unnecessary catheterization. Emerging CCTA techniques like FFR-CT and CT-perfusion show promise but require further clinical validation. Cardiac magnetic resonance (CMR) represents the reference imaging technique for myocardial tissue characterization, useful for distinguishing ischemic from non-ischemic damage. Its limited availability makes it an imaging modality poorly used in the emergency setting, but it plays a fundamental role for a temporally deferred evaluation aimed to reveal the nature and the extension of myocardial injury in selected patients. Advanced CMR techniques (mapping) help in providing more precise diagnosis. Stress Imaging could be indicated for intermediate-risk patients without ECG changes and normal troponin levels. It includes stress-echocardiography, SPECT, PET, and stress CMR. While CCTA has largely replaced these tests, functional imaging remains valuable in specific cases with technical limitations or contrast agent allergies. A more extensive discussion of second-level techniques in the context of ACP can be found in the supplementary materials (supplementary 5.1, 5.2 and 5.3).

## Clinical-diagnostic pathways for patients with ACP

### Risk classification in ACS

Based on the initial ECG and markers of myocardial necrosis, patients with suspected ACS are categorized as follows:

- Patients with ACP (or equivalent symptoms) and persistent ST-segment elevation (or equivalent ST-segment elevation patterns) on ECG: STEMI.
- Patients with ACP (or equivalent symptoms) but without persistent ST-segment elevation (or equivalent ST- segment elevation patterns) on ECG, with elevated and/or decreased cTn levels, with at least one value above the 99th percentile of the upper reference limit: NSTEMI.
- Patients with ACP in the absence of signs of acute cardiomyocyte damage/necrosis, accompanied by specific clinical symptoms: prolonged angina (> 20 min) at rest; new-onset severe angina; increasing frequency or duration of angina, or angina occurring with minimal exertion; or angina occurring after a recent AMI: unstable angina.

### STEMI and high/very high-risk NSTEMI

Within the setting of ACP, prompt and accurate identification of high-risk patients is crucial, as missed diagnoses and inappropriate discharges have been linked to a high mortality rate (2-4%). It is essential to promptly identify ACS patients and establish different pathways for NSTEMI and STEMI. An initial evaluation should consider the patient’s risk factors, as suggested by the latest ESC guidelines [7]. The likelihood that ACP has cardiac origin increases in the presence of multiple risk factors, such as a family history of premature cardiovascular atherosclerotic disease, familial hypercholesterolemia, smoking, hypertension, diabetes mellitus, dyslipidemia, obesity, and kidney failure. Based on these cardiovascular risk factors, patients can already be classified into high and low-risk categories. The risk assessment and initial ECG findings will guide the rapid referral of STEMI patients or high/very high-risk NSTEMI patients for revascularization (Table 1). As suggested by international guidelines [7], it is essential to minimize delays and ensure the ideal time intervals for diagnosis and revascularization. The AHA/ACC consensus document outlines all recommendations for managing patients with ACP and suspected ACS [2]. For STEMI patients, revascularization should be performed as soon as possible [7]. Specifically, immediate primary percutaneous coronary intervention is highly recommended for patients with symptom onset within 12 hours and should be performed within 60 minutes in centers with 24/7 interventional cardiology service and within 90 minutes for patients admitted to spoke centers or transported by ambulance. For NSTEMI patients with any high-risk features, an early invasive strategy (within 24 hours) should be considered, including invasive coronary angiography (ICA) with possible angioplasty within 24 hours of diagnosis [7].

**Table 1:** Criteria for High-Risk and Very High-Risk NSTEMI

*Very-High-Risk NSTEMI*
Hemodynamic instability or cardiogenic shock
Recurrent/ongoing chest pain refractory to medical therapy
Cardiac arrest after presentation or life-threatening arrhythmias
Mechanical complications
Acute heart failure due to ongoing ischemia
Recurrent dynamic ECG changes suggestive of ischemia, especially intermittent ST-segment elevation
*High-Risk NSTEMI*
NSTEMI confirmed by recommended hs-cTn algorithms
Dynamic ST-segment and T-wave changes
Transient ST-segment elevation
GRACE risk score > 140

*ECG* electrocardiogram; *NSTEMI* Non-ST-elevation myocardial infarction; *hs-cTn* high-sensitivity cardiac troponin; *GRACE* Global Registry of Acute Coronary Events

For NSTEMI patients requiring the exclusion of obstructive CAD, CT may play a role only if the local organizational model allows CCTA to be available before ICA, without causing delays in the angiography itself. In an organizational model with prompt availability of CCTA, CCTA can be performed before ICA to reduce the number of unnecessary catheterizations.

The setting of presumptive diagnosis of Acute Aortic Syndrome (AAS) and Pulmonary Embolism (PE) is reported in the supplementary materials (supplementary 6.2.1).

### Non-high risk and uncertain diagnosis

The term “non-high risk” refers to patients who do not show clear signs of AMI or hemodynamic instability and who have a low-to-intermediate probability of ACS [7, 9]. This category includes patients with ACP of possible cardiac origin but without a definitive clinical, electrocardiographic, or laboratory diagnosis of ACS [10, 11]. These patients are often diagnosed with conditions like acute myocarditis, Takotsubo syndrome, or, less commonly, an acute presentation of an unrecognized non-ischemic cardiomyopathy. However, a variable percentage of these patients may have ACS without the characteristics of high-risk STEMI or NSTEMI. In such cases, a preliminary evaluation using CCTA can be a valid alternative to immediate ICA [9]. Comprehensive clinical assessment, including evaluation of diabetes status and duration, is essential, as long-standing diabetes (over 10 years) is associated with a high likelihood of significant coronary calcifications, which could reduce the accuracy of CCTA [12].

**Fig. 3:**
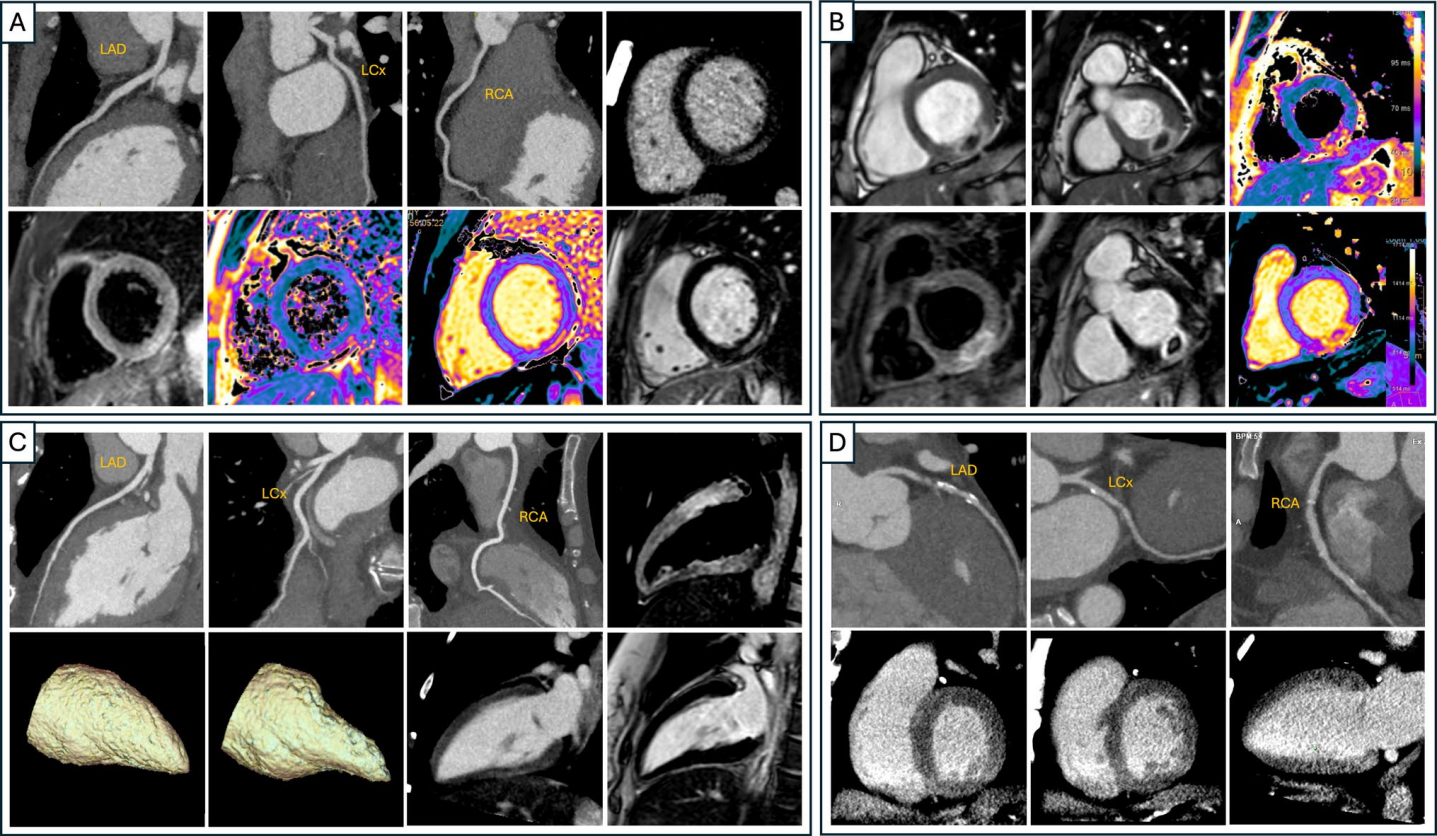
Clinical Cases of Acute Chest Pain. Panel A: Imaging findings of acute infarct-like myocarditis in a 20-year-old male. The first three images (top row) are curved multiplanar reconstructions (cMPRs) of the left anterior descending, circumflex, and right coronary arteries from coronary computed tomography angiography (CCTA), all showing no obstructive coronary disease. The fourth image on the top row is a short-axis late iodine enhancement (LIE) reconstruction highlighting subepicardial enhancement in the inferolateral wall, suggestive of myocardial injury. The bottom row depicts the corresponding cardiovascular magnetic resonance (CMR) findings: short-axis T2 short-tau inversion-recovery (T2-STIR) image demonstrating regional myocardial edema; color-coded quantitative T2 map showing increased T2 values; color-coded native T1 map showing elevated native T1; and late gadolinium enhancement (LGE) image confirming subepicardial necrosis in the same distribution. Panel B: Imaging findings of myocardial infarction with non‑obstructive coronary arteries (MINOCA) in a 36‑year‑old male. The top row presents two cardiac phases (diastolic and systolic) of short‑axis cine steady‑state free precession (cine‑SSFP) illustrating akinesia of the basal‑inferolateral wall, followed by a quantitative T2 map. The T2 map shows a central hypo‑intense core consistent with intramyocardial hemorrhage, surrounded by a rim of increased T2 values indicating myocardial edema. The bottom row depicts short‑axis T2 short‑tau inversion‑recovery (T2‑STIR) image confirming myocardial edema with a central dark zone (hemorrhage); late gadolinium enhancement (LGE) image demonstrating a transmural enhancement pattern with a central hypo‑enhanced region of microvascular obstruction; and a native T1 map showing elevated T1 values within the infarcted myocardium. Panel C: Imaging findings of reverse Takotsubo cardiomyopathy in a 40‑year‑old female. The top row shows curved multiplanar reconstructions (cMPRs) of the left anterior descending (LAD), left circumflex (LCx), and right coronary arteries (RCA) from coronary computed tomography angiography (CCTA), all demonstrating patent, non‑obstructive coronaries, followed by a left‑ventricular long‑axis T2 short‑tau inversion‑recovery (T2‑STIR) image depicting diffuse myocardial edema that predominates in the basal segments. The bottom row includes diastolic and systolic three‑dimensional blood‑pool volume‑rendered models illustrating basal akinesia with compensatory apical hyperkinesia; a long‑axis late iodine enhancement (LIE)/late gadolinium enhancement (LGE) image showing no evidence of myocardial necrosis. Panel D: Imaging findings of non‑ST‑elevation myocardial infarction (NSTEMI) in a 44‑year‑old male (peak high-sensitivity troponin T: 287 ng/L). The top row depicts curved multiplanar reconstructions (cMPRs) of the left anterior descending (LAD), left circumflex (LCx), and right coronary arteries (RCA) from coronary computed tomography angiography (CCTA). A mixed‑attenuation plaque with ulceration causing severe stenosis is evident in the proximal LAD, whereas the LCx and RCA are free of significant disease. The bottom row shows two short‑axis (basal and mid‑ventricular) and one long‑axis late iodine enhancement (LIE) images. All demonstrate absence of LIE, indicating no established myocardial necrosis despite the culprit LAD lesion. *Abbreviations* cMPRs, Curved multiplanar reconstructions; CCTA, Coronary Computed Tomography Angiography; LIE, Late Iodine Enhancement; STIR, Short-TI Inversion Recovery; LGE, Late gadolinium enhancement; MINOCA, Myocardial Infarction with Non-Obstructive Coronary Arteries; SSFP, Steady-state free precession; NSTEMI: Non-ST- elevation myocardial infarction

This information, combined with local factors such as the type of CT scanner available and the expertise of cardiovascular radiologists in interpreting CCTAs, can influence the decision to use CT in the diagnostic workup of a patient with more than 10 years of history of diabetes and ACP. In patients with ACP and low-to-intermediate risk but with unknown CAD, CCTA plays a critical role in ruling out obstructive CAD (stenosis >50%) and in characterizing the atherosclerotic coronary substrate [9]. A diagnostic strategy involving CCTA within 24 hours (preferably within 8 hours to optimize ED workflow and patient experience) provides non-invasive access to coronary anatomical assessment, including the detection of coronary anomalies. CCTA is highly sensitive for both diagnosing obstructive CAD and detecting non- obstructive CAD, which can guide optimized clinical management after the acute phase (Fig. 3). The CCTA use in the ED also allows the exclusion of alternative diagnoses (such as AD or PE), which can sometimes be unexpected. Additionally, when integrated with a multiparametric approach, including a LCE/LIE scan, CCTA allows not only a rapid rule-out of significant CAD or identification of patients who need revascularization but also a prompt differential diagnosis such as myocarditis, MINOCA, or acute cardiomyopathies, all within a “one-stop-shop” approach [13] (Fig. 3). However, the interpretation of LCE/LIE still requires considerable expertise [14], awaiting the broader availability of CT scanners with spectral cardiac imaging capabilities, which could significantly improve LCE/LIE quality and contrast-to-noise ratios. In patients with non-obstructive CAD or normal coronary arteries, the use of CCTA is likely to facilitate early discharge and prevent unnecessary ICA. A more extensive discussion can be found in the supplementary materials (supplementary 5.1).

### Low risk of CAD and presumptive diagnosis of peri-myocarditis or myo-pericarditis

Patients presenting with low-risk ACP (negative ECG for ischemic changes, negative cTn levels, and resolved chest pain) but with known or suspected CAD based on clinical evaluation or pre-test probability scores may be discharged with a recommendation for short-term functional or anatomical imaging tests [5, 9]. Both strategies demonstrate high negative predictive value for the diagnosis of ischemia (functional imaging tests: stress echocardiography [15], SPECT [16], stress CMR [17] and obstructive CCTA [18]). However, in patients without previous anatomical coronary studies (invasive or non-invasive), CCTA is preferable to functional imaging strategies because it can also identify non- obstructive CAD. This enables better prognostic stratification and optimization of therapeutic approaches, positively impacting on long term outcomes [19].

Patients with ACP and no ECG signs suggestive of ischemic changes represent a significant subset of patients with troponin dispersion [20]. In these cases, troponin elevation may be due to various cardiac or non-cardiac causes. Based on the enzyme curve, these patients can be classified as having acute or chronic myocardial injury [21]. For patients with acute myocardial injury, even in the absence of ECG ischemic criteria, it is recommended to rule out obstructive CAD, preferably using CCTA [22, 23]. If the test is negative, CMR is recommended either during hospitalization or with short- term priority to identify myocardial causes of acute troponin dispersion, such as stress cardiomyopathy [24], MINOCA [25], or acute myocarditis [26] (Fig. 3). CMR should be performed as soon as possible, as its diagnostic sensitivity decreases over time, in the days or weeks after the acute event [27].

As previously mentioned, recent studies have proposed CCTA with LCE/LIE as a valid alternative to CMR, capable of providing all necessary information in a single exam [28], thereby improving organizational efficiency and patient safety.

For patients with chronic myocardial injury, CAD evaluation can follow the timing used for low-risk patients. In cases with suspected dilated, inflammatory, infiltrative, or hypertrophic cardiomyopathy (based on clinical, echocardiographic, or laboratory findings), CMR may be indicated to confirm the diagnosis [9]. A more extensive discussion can be found in the supplementary materials (supplementary 5.1 and 5.2).

### Very low risk of CAD and presumptive diagnosis of non-cardiac chest pain

**Fig. 4:**
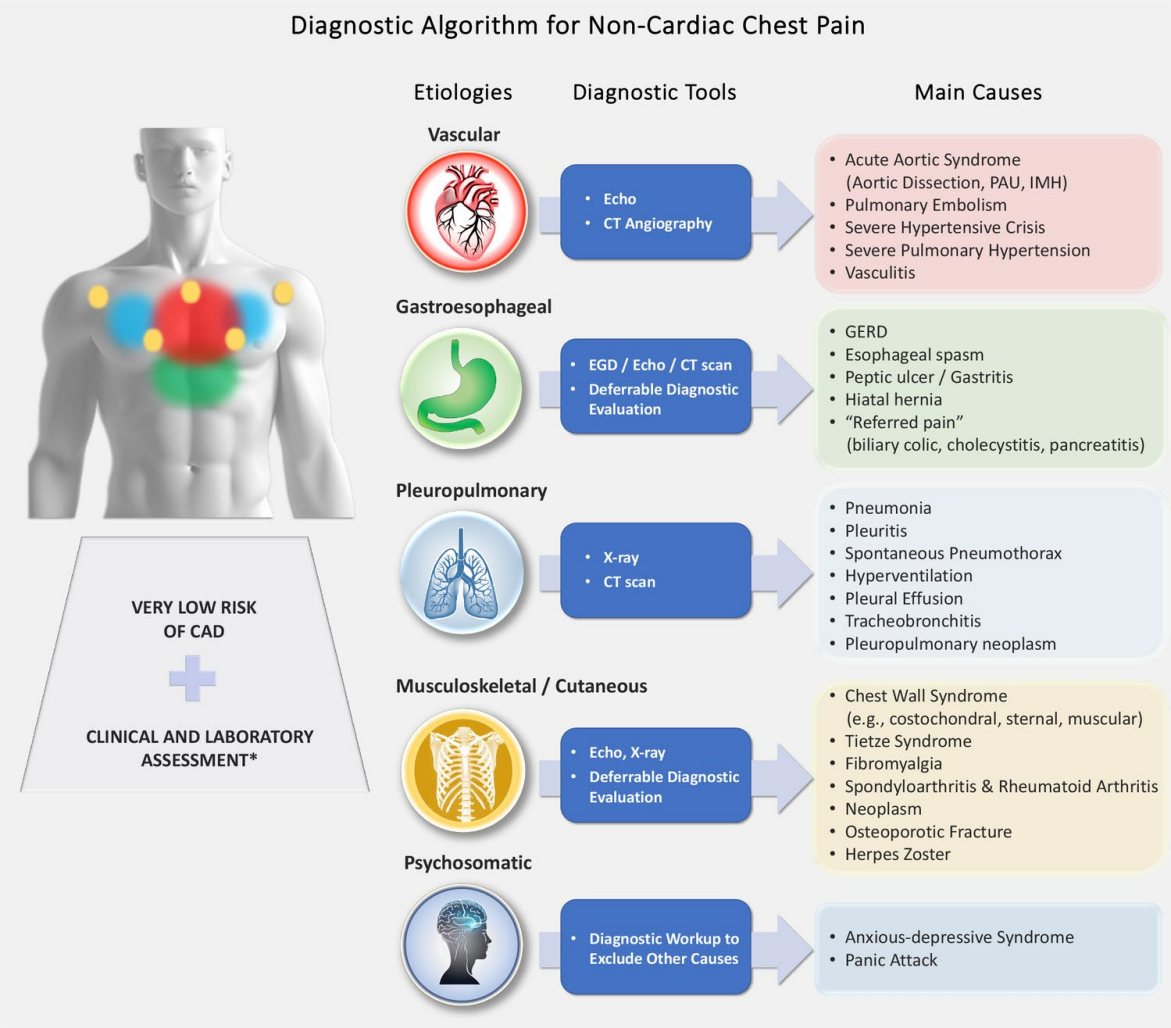
Diagnostic Algorithm for Non-Cardiac Chest Pain. *Abbreviations* CAD, coronary artery disease; CT, computed tomography; EGD, esophagogastroduodenoscopy; GERD, gastroesophageal reflux disease; IMH, intramural hematoma; PAU, penetrating atherosclerotic ulcer; X-Ray, radiography; Echo, Ultrasound as appropriate (e.g., cardiac, abdominal, vascular, musculoskeletal). *: includes the measurement of cardiac biomarkers and D-dimer, complete blood count, and inflammatory markers

In patients where clinical evaluation indicates a very low risk of cardiovascular chest pain, non-cardiac causes must be considered. These may range from benign self-limiting conditions to severe syndromes requiring prompt recognition and timely treatment. Clinical evaluation, integrated with laboratory tests, plays a key role in guiding differential diagnoses. Imaging orders should be tailored to the individual patient, guided by clinical presentation (especially symptoms), vital signs, and laboratory tests. PE can present with few or mild symptoms. When suspected, elevated D- dimer levels warrant CTPA. AAS may manifest as persistent, non-specific chest pain unrelieved for hours, requiring echocardiographic and/or CT evaluation. A more extensive discussion can be found in the supplementary materials (supplementary 5.1). “Non-cardiovascular chest pain” often originates from other thoracic structures, such as the pleura, lungs, esophagus, or chest wall (skin, muscles, or skeleton). Additionally, pain may be “referred” from abdominal organs like the stomach, gallbladder, or pancreas. Proper symptom interpretation based on pain characteristics (acute, constrictive, burning, or dull), location (anterior, posterior, shoulder, radiating to arms or neck), or evolution (relieved by rest, associated with nausea, sweating, fever, etc.) and a thorough physical examination to identify associated signs of a pathological process are essential for guiding diagnostic workup and necessary tests. Low-impact imaging tools such as chest X-ray, TTE, or abdominal ultrasound may be valuable depending on the primary clinical suspicion. Specific considerations for potential non-cardiac causes of chest pain (Fig. 4) include:

- Lungs: Conditions such as PE (potentially life-threatening), pneumonia, pleuritis, or pneumothorax.
- Esophagus: Commonly inflammatory causes. Gastroesophageal reflux disease can mimic angina, while esophagitis may result from drug therapy and should always be investigated. Esophageal motility disorders may also cause pain.
- Chest Wall: Non-traumatic causes include muscle and cartilage inflammation (costochondritis), rheumatic diseases, systemic inflammatory conditions, or rarer causes like fibromyalgia or infectious conditions like herpes zoster. Pain may also indicate primary or secondary osseous or muscular neoplasms, typically localized. Radiographic or ultrasound imaging helps pinpoint the lesion and guide further diagnostic steps.
- Psychosomatic Pain: Panic attacks can mimic cardiac chest pain.
- Referred Pain from Abdominal Causes: Gastrointestinal conditions such as peptic ulcers, biliary colic, or pancreatitis may present as chest pain.

## Organizational and logistical aspects

**Fig. 5:**
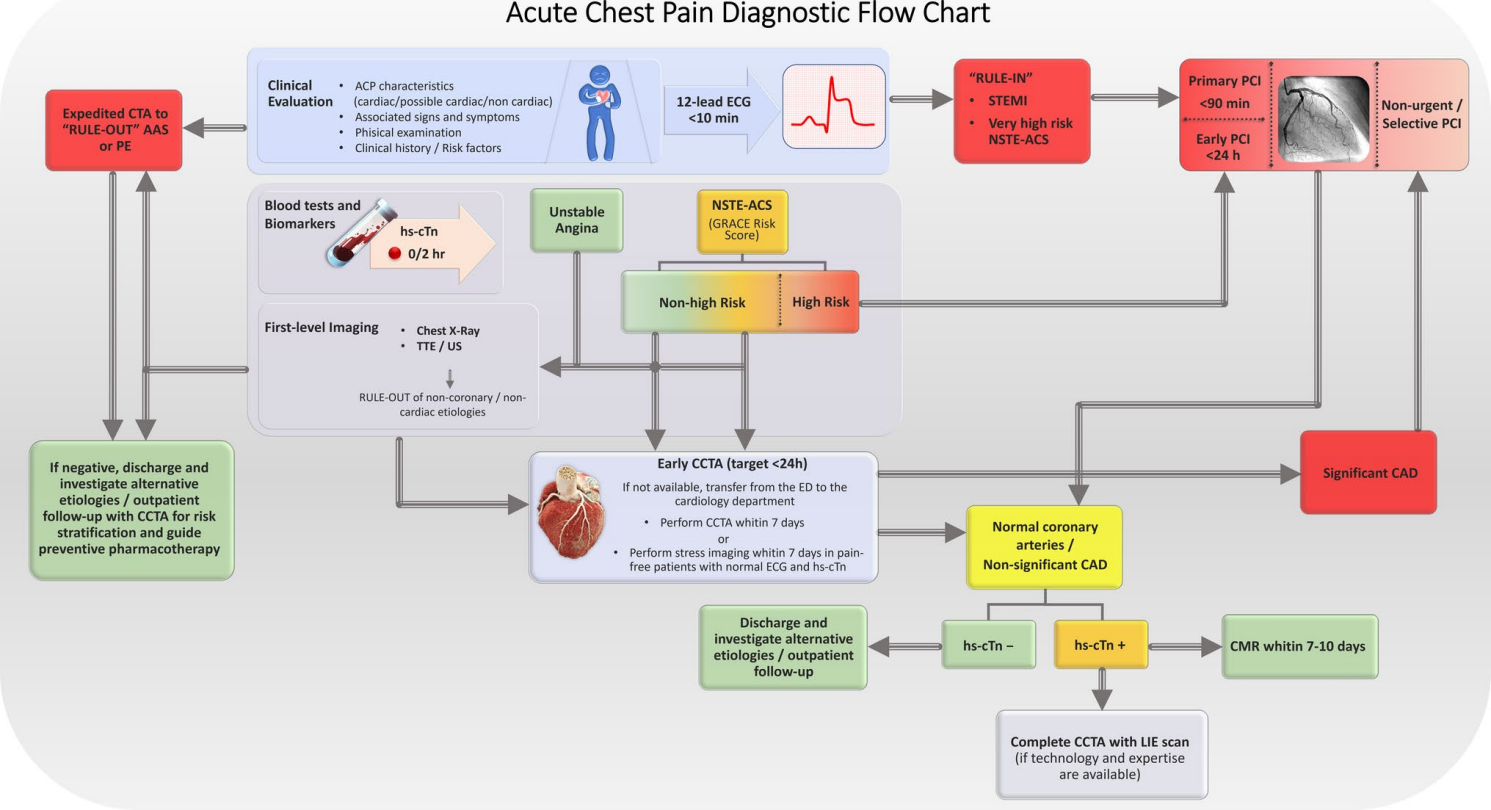
Acute Chest Pain Diagnostic Flow Chart. Blood tests include but are not limited to complete blood count, renal profile, high-sensitivity cardiac troponins (hs-cTn), C-reactive protein (CRP), D-dimer, and NT-pro BNP. Risk stratification for patients with NSTE-ACS is based on the elements described in the 2023 ESC Guidelines [7]. *Abbreviations* AAS, acute aortic syndrome; ACP, acute chest pain; CAD, coronary artery disease; CCTA, coronary computed tomography angiography; CMR, cardiac magnetic resonance; ECG, electrocardiogram; ED, emergency department; GRACE, Global Registry of Acute Coronary Events; hs-cTn, high-sensitivity cardiac troponin; LIE, late iodine enhancement; NSTE-ACS, non-ST-elevation acute coronary syndrome; PCI, percutaneous coronary intervention; PE, pulmonary embolism; X-Ray, radiography; STEMI, ST-elevation myocardial infarction; TTE, transthoracic echocardiography; UA, unstable angina; US, Ultrasound

Collaboration among different specialists, primarily cardiologists and radiologists, is essential to optimise the diagnostic and therapeutic pathway of patients with ACP in the ED. Key challenges include:

- Shared diagnostic pathways (Fig. 5) to be implemented in the clinical practice.
- Availability of advanced imaging. Advanced radiological imaging, in particular CCTA, plays a key role in the management of a sub-population of subjects presenting to ED with ACP. However, the clinical context of subjects with ACP requiring management including CT evaluation, determines the need for CT technology and expertise to address immediately, 24 h a day, the possible suspicion of acute aortic syndrome, which in any case requires the acquisition of CT with cardiosynchronisation for the correct evaluation of the aortic root, and the need for cardioradiological expertise capable of handling all phases of preparation, acquisition and reporting of a cardiac CT examination within 24 h of the arrival of the subject with ACP in the ED.Hence, a state of the art CT scanner with cardiac synchronization capabilities should be always available for patients from ED, as well as a general radiologist able to diagnostically assess patients with suspected AAS. The cardiac CT for ACP can be deferred up to 24-h, hence different delivery strategies can be implemented according to local organisational context: (a) all radiologists providing emergency-service trained to perform advanced cardiac imaging, which is certainly the more complex and difficult to implement in many contexts; (b) deferred execution, within 24 h, of cardiac CT by the cardiovascular CT imaging service in contexts where there is a group of radiologists dedicated to cardiac imaging, associated to the activation of a weekend on-call service; (c) activation of teleradiology support services for emergency rooms in smaller and peripheral settings, through the creation of cardiac imaging networks on a territorial, regional or national basis.
- All the strategies cited above are supported by the adequate training in cardiovascular imaging that radiologists receive during residency and by the continuous training initiatives available, like those by SIRM’s Cardioradiology section [29].
- Outpatient Pathways for advanced cardiac imaging. In some cases deferred follow-up with advanced cardiac imaging could be indicated after ED dismission, hence specific pathways allowing these patients to access the necessary diagnostic method in the days or weeks following their access to the ED, should be impemented.

Organizational and logistical aspects are further addressed in the supplementary materials.

A more extensive discussion of organizational and logistical aspects can be found in the supplementary materials (supplementary 7.1). The central illustration (Fig. 5) presents a suggested approach based on currently available evidence and the insights of experts involved in drafting the document.

## Conclusions

This SIRM-SIC document outlines the appropriate management of patients presenting to the ED with ACP, emphasizing the timing and utility of non-invasive imaging for rapid and accurate diagnosis of cardiac or non-cardiac causes. The document aims to improve diagnostic pathways across Italian hospitals and foster cardiologist-radiologist collaboration, improving patient safety, outcomes, and resource utilization efficiency.

## Supplementary Information

Below is the link to the electronic supplementary material.

Supplementary file1 (DOCX 110 KB)

Supplementary file2 (DOCX 27 KB)

## Abbreviations

AAS: Acute aortic syndrome
ACP: Acute chest pain
ACS: Acute coronary syndrome
AD: Aortic dissection
AMI: Acute myocardial infarction
CAD: Coronary artery disease
CCTA: Coronary computed tomography angiography
CMR: Cardiac magnetic resonance
CXR: Chest radiograph
CTPA: Computed tomography pulmonary angiography
ECG: Electrocardiogram
ECV: Extracellular volume
ED: Emergency department
hs-cTnI: High-sensitivity cardiac troponin I
hs-cTnT: High-sensitivity cardiac troponin T
ICA: Invasive coronary angiography
LCE: Late contrast enhancement
LIE: Late iodine enhancement
MINOCA: Acute myocardial infarction with non-obstructive coronary arteries
NSTEMI: Non-ST-elevation myocardial infarction
PE: Pulmonary embolism
PET: Positron emission tomography
SPECT: Single photon emission computed tomography
STEMI: ST-elevation myocardial infarction
TTE: Transthoracic echocardiography
TRO: Triple rule out
UA: Unstable angina
