# Supplementary material for: SIRM/SIC consensus document on the management of patients with acute chest pain
Source: Radiol Med. 2025 Sep 23;130(12):1936–48. doi: 10.1007/s11547-025-02076-x (PMC12669346; doi:10.1007/s11547-025-02076-x)
Supplement: Supplementary file 1 — Supplementary file1 (DOCX 110 KB) [file 11547_2025_2076_MOESM1_ESM.docx]

**3.4.1 First-line Imaging: Chest Radiography (CXR)**

CXR is a first-line diagnostic exam that is cost-effective, rapidly performed, and available in all ED. It is routinely requested during the initial evaluation of most patients presenting with ACP [^1^], particularly in the absence of a STEMI diagnosis. CXR should ideally be performed within 30 minutes of hospital admission [^2^]. CXR is indicated in patients with atypical presentations of ACS, signs and symptoms of heart failure, or suspected lung disease [^3^]. The only major contraindication to obtaining a CXR in patients with ACP is the potential for delaying primary percutaneous coronary intervention in patients with STEMI [^4^].

CXR can identify signs of heart failure, such as:

• cardiac silhouette enlargement,

• signs of pulmonary congestion (apical redistribution, peribronchial cuffing, Kerley lines, thickened interlobar

fissures, and alveolar edema with a "batwing" distribution),

• and pleural effusion.

All these findings are helpful for diagnostic evaluation and subsequent clinical management, particularly in patients with a history of heart failure. CXR can also detect or suggest various non-cardiac causes of ACP, including pneumonia, evolving pulmonary lesions, pneumothorax, pneumomediastinum, and esophageal rupture [^5^].

2022 ACC/AHA American guidelines still recognize the role of CXR in suspected acute aortic syndrome (AAS), although they acknowledge its significantly lower diagnostic accuracy compared to second-line techniques.

Typical radiographic signs suggestive of AAS include:

• Widening of the mediastinal silhouette,

• Loss of the aortic contour,

• Migration of intimal calcifications,

• A “double density” appearance of the aorta [^6^].

**3.4.2. First-line Imaging: Transthoracic Echocardiography (TTE)**

TTE is the primary non-invasive imaging modality in the setting of suspected ACS. It is safe and repeatable, without contraindications, making it an integral part of the ACP diagnostic algorithm [^7–10^]. Like other imaging methods, TTE must be performed by a skilled operator with an appropriate level of training and expertise [^1^], adhering to the recommendations of major international societies [^7–11^]. In patients with ACP, TTE should include a comprehensive, sequential analysis of standard imaging views (parasternal, apical, subcostal, and suprasternal). In urgency/emergency settings, a focused TTE may be performed, involving a limited set of views to provide a qualitative or semi-quantitative evaluation, often yielding binary (yes/no) answers to specific clinical questions. There are several known or suspected conditions in which TTE is recommended in patients with ACP [^8–12^]. In particular:

1. **ACS:** in suspected acute myocardial ischemia with non-diagnostic ECG and cardiac enzymes, TTE assesses

biventricular function and identifies regional wall motion abnormalities. It may also suggest stress cardiomyopathy (Tako-Tsubo) [^2,5,13,14^].

1. **Hemodynamic instability/shock unresponsive to initial therapies:** TTE rapidly identifies or excludes underlying cardiac causes [^8,13^].
2. **AAS:** TTE may represent the initial imaging modality for detecting intimal flaps (although with low negative predictive value), aneurysms, thrombi, and possible complications such as aortic regurgitation, cardiac tamponade, or wall motion abnormalities [^8,15,16^]. TEE offers superior diagnostic and prognostic accuracy in this setting.
3. **Myocarditis:** despite his limited specificity, TTE can detect systolic and diastolic dysfunction, wall motion abnormalities, wall thickening, reduced Global Longitudinal Strain (GLS) [^17^], and associated pericardial effusion.
4. **Pericarditis:** TTE supports clinical suspicion identifying pericardial effusion and pericardial thickening (>3 mm). In traumatic chest injury, it also rules out pericardial bleeding [^8,18^].
5. **Aortic valve stenosis and hypertrophic cardiomyopathy:** TTE provides a definitive diagnosis and assesses severity.
6. **Pulmonary embolism (PE):** in hemodynamically unstable patients with suspected PE, TTE is a fundamental examination. It detects specific signs of right ventricular dysfunction (e.g., 60/60 sign, McConnell's sign, or visible thrombi) and guides subsequent diagnostic and therapeutic steps [^19,20^].

**4.1 Risk Stratification Using Clinical and Laboratory Data-Based Algorithms**

The HEART (History, ECG, Age, Risk factors, Troponin) score and EDACS (Emergency Department Assessment of Chest Pain Score) score are the most commonly used scoring systems, enabling early discharge of patients due to the low probability of short-term adverse events [^21,22^]. Additionally, the T-MACS (Troponin-only Manchester Acute Coronary Syndromes) score, North American Chest Pain Score, and Vancouver Chest Pain Score are available but less frequently utilized. When ACS is confirmed, the GRACE (Global Registry of Acute Coronary Events) score guides the timing of reperfusion strategies, while the Killip score defines the patient's compensation and hemodynamic status [^23^].

The Wells and Revised Geneva scores assess the probability of PE (Pulmonary Embolism), while the PESI (Pulmonary Embolism Severity Index) stratifies its clinical severity and guides treatment. The PERC (Pulmonary Embolism Rule-out Criteria) is used in patients with a low probability of PE to rule out the diagnosis [^19^].

The AAD (Acute Aortic Dissection) score evaluates the likelihood of acute AD: values above 1 require further specific diagnostic evaluations [^24^]. A description of the risk scores is reported in the supplementary Table 1 (Table S1) [^25–45^].

**5.1. Coronary CT angiography (CCTA)**

The 2021 AHA/ACC/ASE/CHEST/SAEM/SCCT/ SCMR Guideline, the 2022 ESC position paper and the 2024 ESC Guidelines for the management of chronic coronary syndromes [^46–48^] assigned the Class I recommendation, Level of Evidence A, for the use of CCTA as an alternative to angiography in patients with low-to-intermediate risk of CAD and normal or inconclusive findings on cardiac troponins and/or ECG. More recently, the 2023 ESC Guidelines [^23^] for the management of ACS have classified CCTA as Class IIa (Level of Evidence A) in the initial work-up of patients with suspected ACS without recurrent chest pain, normal (or inconclusive) hs-cTn levels, and normal ECG. This update reflects findings from randomized multicenter trials conducted in the era of high-sensitivity cardiac troponin (hs-cTn) testing, which are highly relevant to current clinical practice. The high negative predictive value of CCTA allows to rule out obstructive CAD with high confidence, allowing the safe discharge of patients with ACP. CCTA reduces ED length of stay and improves short- and long-term prognostic risk stratification, also providing cost reductions [^49–51^]. Additionally, in urgent but deferrable scenarios, CCTA is safer, more cost-effective, and more accurate than stress scintigraphy [^52,53^]. According to the recent SIRM-SIC expert consensus [^52^], urgent CCTA is recommended (Level of Evidence B) for patients with low-to-intermediate risk of ACS and inconclusive troponin and ECG results. However, the widespread availability of hs-cTn in triaging patients with ACP reduced the misdiagnosis and the erroneous discharge of patients with ongoing ACS compared to the pre-hs-cTn era and helped to speed up the clinical-laboratory evaluation. Especially in low-risk patients, hs-cTn has improved the safety and speed of discharge. However, a more sensitive test inevitably leads to an increase in false positives results. Consequently, elevated cardiac troponin levels without a clinically evident ACS—a condition termed “troponinosis”—have become increasingly common. Some registries have reported an increase in invasive coronary angiographies and revascularizations of questionable utility linked to the introduction of hs-cTn [^54^]. In this setting, CCTA plays an additional role, being a non-invasive test used to safely and early discharge low-risk patients with negative troponin, but also a valuable method for discriminating low-to-intermediate risk patients with positive hs-cTn without clinical-laboratory signs of STEMI, improving the diagnostic workup and reducing the number of unnecessary catheterizations.

The Hs-cTn also identifies minor myocardial injuries but does not identify their cause. In clinical practice, it can be complex to distinguish dynamic troponin changes caused by NSTEMI from other cardiac and non-cardiac causes. In this context, CCTA can identify those patients with significant coronary obstruction who may benefit from early revascularization. Although both the BEACON and the RAPID CTCA trials failed to demonstrate a significant reduction in major adverse cardiovascular events with the systematic use of CCTA in this setting [^55,56^], it should be considered that this trial used a CT protocol aimed exclusively at excluding significant CAD, while today, multiparametric protocols with broader scanning volumes seem very promising in this area. Moreover, CT represents the most accurate diagnostic method for the identification and staging of other life-threatening causes of ACP that may enter into differential diagnosis with ACS, such as acute AD and PE, both through a single Triple Rule Out (TRO) acquisition protocol and through targeted acquisitions based on suspicion, providing a comprehensive view of the chest that often allows the investigation of major non-vascular thoracic emergencies [^53^]. Additionally, the recently proposed multiparametric cardiac CT protocol in the setting of ACP has gained interest for its ability to investigate a broad spectrum of cardiac causes of troponinosis [^57–59^]. This protocol employs a retrospectively gated CCTA with tube current modulation and the addition of a delayed scan, either single-energy or spectral. It allows the integration of coronary evaluation with both global and segmental myocardial functional assessment, as well as myocardial tissue characterization data through delayed enhancement and extracellular volume (ECV) analysis. Specifically, the inclusion of the delayed scan, known as Late Contrast Enhancement (LCE) or Late Iodine Enhancement (LIE) CT scan, to the CCTA or TRO protocol significantly improves the diagnostic and informational value of cardiac CT in emergency settings. This is achieved by identifying areas of myocardial damage (LCE), their extent, and pattern [^57^], thereby enabling the identification of alternative etiologies to ACS, such as acute myocarditis or AMI with non-obstructive coronary arteries (MINOCA) [^57–59^]. Furthermore, it can determine the location and severity of ischemic damage in patients with significant obstructive CAD [^57^]. However, the incremental benefit of LIE is mitigated by a few limitations: it modestly increases radiation exposure and requires interpretation by radiologists experienced in both myocardial characterization and coronary assessment. State-of-the-art scanners and advanced expertise are essential for high-quality emergency CT imaging to minimize and accurately interpret common artifacts related to high heart rates, breathing, motion, and/or blooming effects [^58,59^]. Advanced technologies also allow the reduction of the radiation dose and contrast medium dose, mitigating the impact of CCTA [^60–64^]. New CT applications such as FFR-CT and CT perfusion [^65,66^] are currently not used in emergency settings due to a lack of context-specific validation and potential limitations related to prolonged post-processing times and, in the case of FFR-CT, challenges in feasibility and accuracy in patients with severe calcifications and/or high or irregular heart rates [^5,58^].

**5.2.** **Cardiac magnetic resonance (CMR)**

CMR is the gold standard imaging technique for myocardial tissue characterization [^52,66,67^] and is critically important for the differential diagnosis of patients with ACP. Due to its limited availability—particularly in emergency settings—its lengthy acquisition and the requirement for patient cooperation, CMR is exclusively reserved for deferred, second-line evaluations after obstructive CAD exclusion in an emergency context. CMR is the reference imaging modality for the diagnosis of myocarditis and for the diagnostic and prognostic assessment of cardiomyopathies [^68^]. Furthermore, the recent introduction of mapping techniques allows the identification and quantification of abnormalities that are not detectable with conventional methods. Specifically, native T1 is highly sensitive but less specific; it increases in conditions such as edema, hyperemia, cellular injury, fibrosis, and amyloid accumulation, while it decreases in the presence of iron or fat [^69,70^]. T2 mapping is more specific for detecting edema and inflammation [^71^]. The extracellular volume (ECV) fraction, calculated from pre- and post-contrast T1 mapping values using blood (hematocrit) as reference tissue, enables quantification of the myocardial interstitium. Interstitial expansion is primarily associated with acute inflammatory processes, chronic fibrotic remodeling, and amyloid deposition [^72,73^]. Due to inter-scanner variability of T1 relaxation times, establishing local reference values is essential. In the context of dynamic troponin changes, CMR enables differentiation between ischemic injury (AMI and MINOCA) and non-ischemic injury (myocarditis, Takotsubo syndrome, and cardiomyopathies) based on the distribution of myocardial edema and necrosis. Ischemic patterns are characterized by subendocardial or transmural involvement, whereas non-ischemic patterns typically display intramyocardial or subepicardial involvement.

However, CMR may yield negative findings in up to 25% of patients with MINOCA, without definitively excluding an ischemic etiology. Ischemic myocardial injury with minimal troponin release (such as in cases of transient epicardial coronary spasm or plaque erosion with low thrombotic burden and distal embolization) or mild necrosis with a diffuse pattern (such as in cases of microvascular spasm), may result in a falsely negative CMR for ischemic causes [^74^]. Nevertheless, although negative CMR findings may occur in 25% of cases of troponinosis, the absence of pathological findings on CMR is consistently associated with a favorable prognosis, regardless of the underlying cause of the troponinosis.

To maximize its diagnostic and prognostic performance, CMR should ideally be performed within 24-72 hours of symptom onset, including mapping techniques whenever feasible [^47,55^]. Several months after the ischemic or non-ischemic event, a follow-up CMR may be indicated to monitor disease progression and provide a long-term prognostic assessment. CMR follow-up allows the evaluation of edema resolution, the development of fibrotic or fibro-adipose changes, and ventricular remodeling [^75,76^].

**5.3. Stress Imaging**

Intermediate risk patients, in the absence of ECG changes and with normal troponin levels for at least 12-24 hours, can undergo stress imaging during hospitalization or shortly after discharge [^5,77^]. CCTA has generally replaced functional imaging in this context; however, functional imaging may be a valid alternative in specific settings with outdated CT technology and/or limited CCTA expertise, which complicates the evaluation of patients with irregular heart rates, extensive coronary calcifications or prior revascularizations with small stents. Furthermore, functional stress imaging may be considered in cases of severe iodine contrast allergies or significant kidney failure. Available stress imaging techniques include echocardiography, myocardial scintigraphy (Single Photon Emission Computed Tomography - SPECT), positron emission tomography (PET) and stress CMR. CT-perfusion is emerging with extremely promising data. In patients with ACP at intermediate risk of CAD, the combined evaluation of CCTA and CT-perfusion has proven to be accurate in identifying significant stenoses on invasive coronary angiography, with the advantage of lower costs and shorter hospital stays compared to SPECT [^78^]. However, CT-perfusion is not yet considered the clinical standard, particularly in the acute setting. Exercise ECG is currently considered less accurate in detecting ischemia due to its reduced sensitivity and specificity [^79^]. The choice of a specific diagnostic test depends on patient characteristics, operator expertise, technique availability, and local resources. Stress echocardiography is the most widely available and cost-effective method, which can be performed using protocols involving either physical exercise or pharmacological stress [^80^]. However, its diagnostic value is limited in the presence of poor acoustic windows, and its sensitivity is lower compared to other stress imaging tests [^81^]. Several studies have shown that a negative SPECT in patients presenting to the ED with ACP and uncertain diagnosis is associated with an excellent prognosis [^82^]. PET represents the gold standard for myocardial perfusion studies [^83^], but its use is limited by availability. Finally, stress CMR is a highly valuable diagnostic and prognostic technique in patients with ACP [^84^]. In addition to assessing myocardial perfusion, CMR allows tissue characterization, identifying alternative causes of chest pain such as myocarditis, pericarditis, or Takotsubo syndrome [^84,85^]. The main limitation of the widespread application of stress CMR in this setting is its limited availability in the emergency setting.

**6.2.1. Presumptive Diagnosis: Acute Aortic Syndrome (AAS); Pulmonary Embolism (PE)**

AAS and PE are two clinical conditions that can present with ACP and require timely diagnosis. AAS encompasses several conditions that result in a disruption of the aortic wall with an abrupt onset, includingAD, intramural hematoma, and penetrating aortic ulcer. Timely diagnosis and treatment are critical, as these conditions can lead to complete rupture of the aortic wall or severe complications due to organ malperfusion.

PE is the third most common acute cardiovascular syndrome globally, after AMI and stroke. It can be life-threatening if not diagnosed and treated promptly [^86^]. CT plays a key role in the diagnosis and therapeutic management of both conditions. In case of suspected AAS, CT is the diagnostic modality of choice and should be performed as soon as possible. It provides excellent sensitivity and specificity in diagnosing AAS but must be performed and interpreted by expert radiologists using appropriate techniques, such as ECG synchronization, to avoid pulsatility artifacts at the level of the aortic root [^87^]. Additionally, CT allows assessment of coronary involvement and simultaneously excludes other causes of ACP. A multi-phase CT acquisition is necessary, starting with a non-contrast scan to identify any intramural hematomas and accurately identify surgical materials in patients with a history of prior aortic intervention, followed by arterial and venous scans to assess vascular structures and organ ischemia [^6^]. Diagnostic algorithms for suspected PE are divided into two main pathways: PE "with" and "without" hemodynamic instability (cardiac arrest, obstructive shock, and persistent hypotension). For patients with hemodynamic instability, CT pulmonary angiography (CTPA) is recommended for diagnosis if immediately available and feasible. In patients without hemodynamic instability, CTPA is suggested as the first step if there is a high clinical probability or suspected PE. For patients with low or intermediate clinical probability or unlikely PE, CTPA is recommended if the D-dimer test is positive [^88^].

**7.1. Logistical and Organizational Aspects**

The collaboration between cardiologists and radiologists in the ED is essential for improving the diagnostic-therapeutic pathway for patients with ACP. The main challenges to be addressed involve the following points.

Availability and adherence to a shared protocol.

The sharing of a protocol for cardiac patients in the ED often faces considerable difficulties in the acute care setting. Furthermore, the implementation of intervention-based recommendations, such as those outlined in this document, proves more challenging compared to monitoring-only recommendations. Therefore, a shared pathway involving emergency medicine physicians, cardiologists, and radiologists must be implemented and integrated into clinical practice.

Availability of second-level imaging modalities.

A fundamental aspect of adapting the protocol to clinical reality lies in the availability of various radiological exams, which may vary depending on the day of the week. The availability of a radiologist with advanced cardiovascular expertise, capable of fully utilizing CCTA for comprehensive diagnostic purposes, according to the needs defined in this manuscript, is essential in a modern ED.

Development of an outpatient pathway for patients without signs/symptoms of ACS or critical coronary disease.

While hospitalization for patients with a positive second-level imaging test is a well-defined process, the appropriate pharmacological management and further investigations for discharged patients remain highly dependent on individual clinicians, often lacking clear scientific evidence. Proper evaluation of cardiovascular risk factors and the clinical profile of discharged patients is therefore necessary to optimize subsequent therapy.

Adequate radiological equipment and expertise.

The key prerequisites for an effective radiology service capable of managing patients with ACP are appropriate technical equipment and comprehensive training for both medical and technical staff.

The minimum technical standard generally considered sufficient for coronary CT imaging requires a scanner with at least 64 slices, cardiac synchronization software, and an automatic injector for contrast media bolus and saline solution administration. However, in acute care settings, advanced CT scanners exceeding the 64-slice level, with a detector panel width of at least 8 cm along the z-axis, tube-detector rotation times below 300 ms, and/or dual-source technology, are highly desirable to consistently achieve high-quality imaging in the complex setting of ACP in the ED.

Training for radiologists in cardiovascular imaging starts during residency. According to a recent survey conducted by the cardioradiology section of SIRM [^89^], significant efforts have been made in Italy to enhance knowledge of cardiac imaging starting from residency programs. Over the years, SIRM, through its Cardioradiology Section, has developed an extensive educational network to support the growth of this subspecialty, yielding highly positive results and leading to widespread availability of expertise throughout the country [^89^].

Training for technical staff is equally critical and should be supported by the development of well-defined protocols that are easy to select and apply, thereby optimizing and standardizing exam acquisition, particularly in emergency settings.

Lastly, the organizational aspect at the local level is of no less importance. It primarily relies on the creation of a company-level shared diagnostic-therapeutic pathway (PDTA) in collaboration with cardiologists. This protocol must take into account the available equipment and personnel to ensure a coherent response to the requests for advanced imaging in patients with ACP.

**BIBLIOGRAPHY**

1. Zuin G, Parato VM, Groff P, et al. ANMCO-SIMEU Consensus Document: in-hospital management of patients presenting with chest pain. *European Heart Journal Supplements*. 2017;19(suppl_D):D212-D228. doi:10.1093/eurheartj/sux025

2. Stepinska J, Lettino M, Ahrens I, et al. Diagnosis and risk stratification of chest pain patients in the emergency department: focus on acute coronary syndromes. A position paper of the Acute Cardiovascular Care Association. *European Heart Journal: Acute Cardiovascular Care*. 2020;9(1):76-89. doi:10.1177/2048872619885346

3. Knuuti J, Wijns W, Saraste A, et al. 2019 ESC Guidelines for the diagnosis and management of chronic coronary syndromes. *European Heart Journal*. 2020;41(3):407-477. doi:10.1093/eurheartj/ehz425

4. Kontos MC, De Lemos JA, Deitelzweig SB, et al. 2022 ACC Expert Consensus Decision Pathway on the Evaluation and Disposition of Acute Chest Pain in the Emergency Department. *Journal of the American College of Cardiology*. 2022;80(20):1925-1960. doi:10.1016/j.jacc.2022.08.750

5. Collet JP, Thiele H, Barbato E, et al. 2020 ESC Guidelines for the management of acute coronary syndromes in patients presenting without persistent ST-segment elevation. *European Heart Journal*. 2021;42(14):1289-1367. doi:10.1093/eurheartj/ehaa575

6. Isselbacher EM, Preventza O, Hamilton Black J, et al. 2022 ACC/AHA Guideline for the Diagnosis and Management of Aortic Disease: A Report of the American Heart Association/American College of Cardiology Joint Committee on Clinical Practice Guidelines. *Circulation*. 2022;146(24). doi:10.1161/CIR.0000000000001106

7. Popescu (Chair) BA, Stefanidis A, Fox KF, et al. Training, competence, and quality improvement in echocardiography: the European Association of Cardiovascular Imaging Recommendations: update 2020. *European Heart Journal - Cardiovascular Imaging*. 2020;21(12):1305-1319. doi:10.1093/ehjci/jeaa266

8. Lancellotti P, Price S, Edvardsen T, et al. The use of echocardiography in acute cardiovascular care: Recommendations of the European Association of Cardiovascular Imaging and the Acute Cardiovascular Care Association. *European Heart Journal: Acute Cardiovascular Care*. 2015;4(1):3-5. doi:10.1177/2048872614568073

9. Neskovic AN, Hagendorff A, Lancellotti P, et al. Emergency echocardiography: the European Association of Cardiovascular Imaging recommendations. *European Heart Journal - Cardiovascular Imaging*. 2013;14(1):1-11. doi:10.1093/ehjci/jes193

10. Beygui F, Castren M, Brunetti ND, et al. Pre-hospital management of patients with chest pain and/or dyspnoea of cardiac origin. A position paper of the Acute Cardiovascular Care Association (ACCA) of the ESC. *European Heart Journal: Acute Cardiovascular Care*. 2020;9(1_suppl):59-81. doi:10.1177/2048872615604119

11. Galderisi M, Cardim N, D’Andrea A, et al. The multi-modality cardiac imaging approach to the Athlete’s heart: an expert consensus of the European Association of Cardiovascular Imaging. *European Heart Journal - Cardiovascular Imaging*. 2015;16(4):353-353r. doi:10.1093/ehjci/jeu323

12. Galderisi M, Cosyns B, Edvardsen T, et al. Standardization of adult transthoracic echocardiography reporting in agreement with recent chamber quantification, diastolic function, and heart valve disease recommendations: an expert consensus document of the European Association of Cardiovascular Imaging. *European Heart Journal - Cardiovascular Imaging*. 2017;18(12):1301-1310. doi:10.1093/ehjci/jex244

13. Ibanez B, James S, Agewall S, et al. 2017 ESC Guidelines for the management of acute myocardial infarction in patients presenting with ST-segment elevation. *European Heart Journal*. 2018;39(2):119-177. doi:10.1093/eurheartj/ehx393

14. Dahlslett T, Karlsen S, Grenne B, et al. Early Assessment of Strain Echocardiography Can Accurately Exclude Significant Coronary Artery Stenosis in Suspected Non–ST-Segment Elevation Acute Coronary Syndrome. *Journal of the American Society of Echocardiography*. 2014;27(5):512-519. doi:10.1016/j.echo.2014.01.019

15. 2014 ESC Guidelines on the diagnosis and treatment of aortic diseases: Document covering acute and chronic aortic diseases of the thoracic and abdominal aorta of the adultThe Task Force for the Diagnosis and Treatment of Aortic Diseases of the European Society of Cardiology (ESC). *Eur Heart J*. 2014;35(41):2873-2926. doi:10.1093/eurheartj/ehu281

16. Meredith EL, Masani ND. Echocardiography in the emergency assessment of acute aortic syndromes. *European Journal of Echocardiography*. 2009;10(1):i31-i39. doi:10.1093/ejechocard/jen251

17. Caforio ALP, Pankuweit S, Arbustini E, et al. Current state of knowledge on aetiology, diagnosis, management, and therapy of myocarditis: a position statement of the European Society of Cardiology Working Group on Myocardial and Pericardial Diseases. *European Heart Journal*. 2013;34(33):2636-2648. doi:10.1093/eurheartj/eht210

18. Adler Y, Charron P, Imazio M, et al. 2015 ESC Guidelines for the diagnosis and management of pericardial diseases. *European Heart Journal*. 2015;36(42):2921-2964. doi:10.1093/eurheartj/ehv318

19. Konstantinides SV, Meyer G, Becattini C, et al. 2019 ESC Guidelines for the diagnosis and management of acute pulmonary embolism developed in collaboration with the European Respiratory Society (ERS). *European Heart Journal*. 2020;41(4):543-603. doi:10.1093/eurheartj/ehz405

20. Donato Mele, Matteo Cameli, Andrea Fiorencis, et al. Ruolo attuale dell’ecocardiografia nei pazienti con embolia polmonare. *Giornale Italiano di Cardiologia*. 2014;(2014Dicembre). doi:10.1714/1718.18772

21. Stopyra JP, Miller CD, Hiestand BC, et al. Performance of the EDACS-accelerated Diagnostic Pathway in a Cohort of US Patients with Acute Chest Pain. *Critical Pathways in Cardiology: A Journal of Evidence-Based Medicine*. 2015;14(4):134-138. doi:10.1097/HPC.0000000000000059

22. Fiore G, Pinto G, Preda A, et al. Performances of HEART score to predict 6-month prognostic of emergency department patients with chest pain: a retrospective cohort analysis. *European Journal of Emergency Medicine*. 2023;30(3):179-185. doi:10.1097/MEJ.0000000000001022

23. Byrne RA, Rossello X, Coughlan JJ, et al. 2023 ESC Guidelines for the management of acute coronary syndromes. *European Heart Journal*. 2023;44(38):3720-3826. doi:10.1093/eurheartj/ehad191

24. Bima P, Pivetta E, Nazerian P, et al. Systematic Review of Aortic Dissection Detection Risk Score Plus D‐dimer for Diagnostic Rule‐out Of Suspected Acute Aortic Syndromes. Carpenter CR, ed. *Academic Emergency Medicine*. 2020;27(10):1013-1027. doi:10.1111/acem.13969

25. Conti A, Paladini B, Toccafondi S, et al. Effectiveness of a multidisciplinary chest pain unit for the assessment of coronary syndromes and risk stratification in the Florence area. *American Heart Journal*. 2002;144(4):630-635. doi:10.1067/mhj.2002.124352

26. Than M, Flaws D, Sanders S, et al. Development and validation of the E mergency D epartment A ssessment of C hest pain S core and 2 h accelerated diagnostic protocol. *Emerg Medicine Australasia*. 2014;26(1):34-44. doi:10.1111/1742-6723.12164

27. Six AJ, Backus BE, Kelder JC. Chest pain in the emergency room: value of the HEART score. *NHJL*. 2008;16(6):191-196. doi:10.1007/BF03086144

28. Backus BE, Six AJ, Kelder JC, et al. A prospective validation of the HEART score for chest pain patients at the emergency department. *International Journal of Cardiology*. 2013;168(3):2153-2158. doi:10.1016/j.ijcard.2013.01.255

29. Granger CB. Predictors of Hospital Mortality in the Global Registry of Acute Coronary Events. *Arch Intern Med*. 2003;163(19):2345. doi:10.1001/archinte.163.19.2345

30. Eagle KA, Lim MJ, Dabbous OH, et al. A Validated Prediction Model for All Forms of Acute Coronary Syndrome: Estimating the Risk of 6-Month Postdischarge Death in an International Registry. *JAMA*. 2004;291(22):2727. doi:10.1001/jama.291.22.2727

31. Fox KAA, Dabbous OH, Goldberg RJ, et al. Prediction of risk of death and myocardial infarction in the six months after presentation with acute coronary syndrome: prospective multinational observational study (GRACE). *BMJ*. 2006;333(7578):1091. doi:10.1136/bmj.38985.646481.55

32. Fox KAA, FitzGerald G, Puymirat E, et al. Should patients with acute coronary disease be stratified for management according to their risk? Derivation, external validation and outcomes using the updated GRACE risk score. *BMJ Open*. 2014;4(2):e004425. doi:10.1136/bmjopen-2013-004425

33. Wenzl FA, Kraler S, Ambler G, et al. Sex-specific evaluation and redevelopment of the GRACE score in non-ST-segment elevation acute coronary syndromes in populations from the UK and Switzerland: a multinational analysis with external cohort validation. *The Lancet*. 2022;400(10354):744-756. doi:10.1016/S0140-6736(22)01483-0

34. Antman EM, Cohen M, Bernink PJLM, et al. The TIMI Risk Score for Unstable Angina/Non–ST Elevation MI: A Method for Prognostication and Therapeutic Decision Making. *JAMA*. 2000;284(7):835. doi:10.1001/jama.284.7.835

35. Morrow DA, Antman EM, Charlesworth A, et al. TIMI Risk Score for ST-Elevation Myocardial Infarction: A Convenient, Bedside, Clinical Score for Risk Assessment at Presentation: An Intravenous nPA for Treatment of Infarcting Myocardium Early II Trial Substudy. *Circulation*. 2000;102(17):2031-2037. doi:10.1161/01.CIR.102.17.2031

36. Boersma E, Pieper KS, Steyerberg EW, et al. Predictors of Outcome in Patients With Acute Coronary Syndromes Without Persistent ST-Segment Elevation: Results From an International Trial of 9461 Patients. *Circulation*. 2000;101(22):2557-2567. doi:10.1161/01.CIR.101.22.2557

37. Killip T, Kimball JT. Treatment of myocardial infarction in a coronary care unit. *The American Journal of Cardiology*. 1967;20(4):457-464. doi:10.1016/0002-9149(67)90023-9

38. Khot UN, Jia G, Moliterno DJ, et al. Prognostic Importance of Physical Examination for Heart Failure in Non–ST-Elevation Acute Coronary Syndromes: The Enduring Value of Killip Classification. *JAMA*. 2003;290(16):2174. doi:10.1001/jama.290.16.2174

39. Itzahki Ben Zadok O, Ben-Gal T, Abelow A, et al. Temporal Trends in the Characteristics, Management and Outcomes of Patients With Acute Coronary Syndrome According to Their Killip Class. *The American Journal of Cardiology*. 2019;124(12):1862-1868. doi:10.1016/j.amjcard.2019.09.012

40. Wells PS, Anderson DR, Rodger M, et al. Excluding Pulmonary Embolism at the Bedside without Diagnostic Imaging: Management of Patients with Suspected Pulmonary Embolism Presenting to the Emergency Department by Using a Simple Clinical Model and d -dimer. *Ann Intern Med*. 2001;135(2):98. doi:10.7326/0003-4819-135-2-200107170-00010

41. Kline JA, Mitchell AM, Kabrhel C, Richman PB, Courtney DM. Clinical criteria to prevent unnecessary diagnostic testing in emergency department patients with suspected pulmonary embolism. *Journal of Thrombosis and Haemostasis*. 2004;2(8):1247-1255. doi:10.1111/j.1538-7836.2004.00790.x

42. Le Gal G, Righini M, Roy PM, et al. Prediction of Pulmonary Embolism in the Emergency Department: The Revised Geneva Score. *Ann Intern Med*. 2006;144(3):165. doi:10.7326/0003-4819-144-3-200602070-00004

43. Aujesky D, Obrosky DS, Stone RA, et al. Derivation and Validation of a Prognostic Model for Pulmonary Embolism. *Am J Respir Crit Care Med*. 2005;172(8):1041-1046. doi:10.1164/rccm.200506-862OC

44. Rogers AM, Hermann LK, Booher AM, et al. Sensitivity of the Aortic Dissection Detection Risk Score, a Novel Guideline-Based Tool for Identification of Acute Aortic Dissection at Initial Presentation: Results From the International Registry of Acute Aortic Dissection. *Circulation*. 2011;123(20):2213-2218. doi:10.1161/CIRCULATIONAHA.110.988568

45. Nazerian P, Mueller C, Soeiro ADM, et al. Diagnostic Accuracy of the Aortic Dissection Detection Risk Score Plus D-Dimer for Acute Aortic Syndromes: The ADvISED Prospective Multicenter Study. *Circulation*. 2018;137(3):250-258. doi:10.1161/CIRCULATIONAHA.117.029457

46. Pontone G, Rossi A, Guglielmo M, et al. Clinical applications of cardiac computed tomography: a consensus paper of the European Association of Cardiovascular Imaging—part I. *European Heart Journal - Cardiovascular Imaging*. 2022;23(3):299-314. doi:10.1093/ehjci/jeab293

47. Pontone G, Rossi A, Guglielmo M, et al. Clinical applications of cardiac computed tomography: a consensus paper of the European Association of Cardiovascular Imaging—part II. *European Heart Journal-Cardiovascular Imaging*. 2022;23(4):e136-e161.

48. Vrints C, Andreotti F, Koskinas KC, et al. 2024 ESC Guidelines for the management of chronic coronary syndromes. *European Heart Journal*. 2024;45(36):3415-3537. doi:10.1093/eurheartj/ehae177

49. Hoffmann U, Bamberg F, Chae CU, et al. Coronary Computed Tomography Angiography for Early Triage of Patients With Acute Chest Pain. *Journal of the American College of Cardiology*. 2009;53(18):1642-1650. doi:10.1016/j.jacc.2009.01.052

50. Hoffmann U, Truong QA, Fleg JL, et al. Design of the Rule Out Myocardial Ischemia/Infarction Using Computer Assisted Tomography: A multicenter randomized comparative effectiveness trial of cardiac computed tomography versus alternative triage strategies in patients with acute chest pain in the emergency department. *American Heart Journal*. 2012;163(3):330-338.e1. doi:10.1016/j.ahj.2012.01.028

51. Chang HJ, Lin FY, Gebow D, et al. Selective Referral Using CCTA Versus Direct Referral for Individuals Referred to Invasive Coronary Angiography for Suspected CAD. *JACC: Cardiovascular Imaging*. 2019;12(7):1303-1312. doi:10.1016/j.jcmg.2018.09.018

52. Carrabba N, Pontone G, Andreini D, et al. Appropriateness criteria for the use of cardiac computed tomography, SIC-SIRM part 2: acute chest pain evaluation; stent and coronary artery bypass graft patency evaluation; planning of coronary revascularization and transcatheter valve procedures; cardiomyopathies, electrophysiological applications, cardiac masses, cardio-oncology and pericardial diseases evaluation. *Journal of Cardiovascular Medicine*. 2022;23(5):290-303. doi:10.2459/JCM.0000000000001303

53. Eltabbakh AR, Dawoud MA, Langer M, Moharm MA, Hamdy EA, Hamisa MF. ‘Triple-rule-out’ CT angiography for clinical decision making and early triage of acute chest pain patients: use of 320-multislice CT angiography. *Egypt J Radiol Nucl Med*. 2019;50(1):3. doi:10.1186/s43055-019-0003-1

54. Shah ASV, Anand A, Strachan FE, et al. High-sensitivity troponin in the evaluation of patients with suspected acute coronary syndrome: a stepped-wedge, cluster-randomised controlled trial. *The Lancet*. 2018;392(10151):919-928. doi:10.1016/S0140-6736(18)31923-8

55. Gray AJ, Roobottom C, Smith JE, et al. Early computed tomography coronary angiography in patients with suspected acute coronary syndrome: randomised controlled trial. *BMJ*. Published online September 29, 2021:n2106. doi:10.1136/bmj.n2106

56. Dedic A, Lubbers MM, Schaap J, et al. Coronary CT Angiography for Suspected ACS in the Era of High-Sensitivity Troponins. *Journal of the American College of Cardiology*. 2016;67(1):16-26. doi:10.1016/j.jacc.2015.10.045

57. Vignale D, Palmisano A, Colantoni C, et al. Toward a One-Stop Shop CT Protocol in Acute Chest Pain Syndrome. *Radiology*. 2023;306(1):E3-E4. doi:10.1148/radiol.220844

58. Marano R, Rovere G, Savino G, et al. CCTA in the diagnosis of coronary artery disease. *Radiol med*. 2020;125(11):1102-1113. doi:10.1007/s11547-020-01283-y

59. Haase R, Schlattmann P, Gueret P, et al. Diagnosis of obstructive coronary artery disease using computed tomography angiography in patients with stable chest pain depending on clinical probability and in clinically important subgroups: meta-analysis of individual patient data. *BMJ*. Published online June 12, 2019:l1945. doi:10.1136/bmj.l1945

60. Toia P, La Grutta L, Smeraldi T, et al. Updated diagnostic & prognostic paradigm for CAD: a narrative review. *Cardiovasc Diagn Ther*. 2020;10(6):1979-1991. doi:10.21037/cdt-20-526

61. Reinhardt SW, Lin CJ, Novak E, Brown DL. Noninvasive Cardiac Testing vs Clinical Evaluation Alone in Acute Chest Pain: A Secondary Analysis of the ROMICAT-II Randomized Clinical Trial. *JAMA Intern Med*. 2018;178(2):212. doi:10.1001/jamainternmed.2017.7360

62. Agliata G, Schicchi N, Agostini A, et al. Radiation exposure related to cardiovascular CT examination: comparison between conventional 64-MDCT and third-generation dual-source MDCT. *Radiol med*. 2019;124(8):753-761. doi:10.1007/s11547-019-01036-6

63. Schicchi N, Mari A, Fogante M, et al. In vivo radiation dosimetry and image quality of turbo-flash and retrospective dual-source CT coronary angiography. *Radiol med*. 2020;125(2):117-127. doi:10.1007/s11547-019-01103-y

64. Di Cesare E, Patriarca L, Panebianco L, et al. Coronary computed tomography angiography in the evaluation of intermediate risk asymptomatic individuals. *Radiol med*. 2018;123(9):686-694. doi:10.1007/s11547-018-0898-z

65. Kelion AD, Nicol ED. The rationale for the primacy of coronary CT angiography in the National Institute for Health and Care Excellence (NICE) guideline (CG95) for the investigation of chest pain of recent onset. *Journal of Cardiovascular Computed Tomography*. 2018;12(6):516-522. doi:10.1016/j.jcct.2018.09.001

66. Pontone G, Di Cesare E, Castelletti S, et al. Appropriate use criteria for cardiovascular magnetic resonance imaging (CMR): SIC—SIRM position paper part 1 (ischemic and congenital heart diseases, cardio-oncology, cardiac masses and heart transplant). *Radiol med*. 2021;126(3):365-379. doi:10.1007/s11547-020-01332-6

67. Barison A, Baritussio A, Cipriani A, et al. Cardiovascular magnetic resonance: What clinicians should know about safety and contraindications. *International Journal of Cardiology*. 2021;331:322-328. doi:10.1016/j.ijcard.2021.02.003

68. Kramer CM, Barkhausen J, Bucciarelli-Ducci C, Flamm SD, Kim RJ, Nagel E. Standardized cardiovascular magnetic resonance imaging (CMR) protocols: 2020 update. *Journal of Cardiovascular Magnetic Resonance*. 2020;22(1):17. doi:10.1186/s12968-020-00607-1

69. Bulluck H, Maestrini V, Rosmini S, et al. Myocardial T1 Mapping: – Hope or Hype? –. *Circ J*. 2015;79(3):487-494. doi:10.1253/circj.CJ-15-0054

70. Radenkovic D, Weingärtner S, Ricketts L, Moon JC, Captur G. T1 mapping in cardiac MRI. *Heart Fail Rev*. 2017;22(4):415-430. doi:10.1007/s10741-017-9627-2

71. Verhaert D, Thavendiranathan P, Giri S, et al. Direct T2 Quantification of Myocardial Edema in Acute Ischemic Injury. *JACC: Cardiovascular Imaging*. 2011;4(3):269-278. doi:10.1016/j.jcmg.2010.09.023

72. Kellman P, Wilson JR, Xue H, Ugander M, Arai AE. Extracellular volume fraction mapping in the myocardium, part 1: evaluation of an automated method. *Journal of Cardiovascular Magnetic Resonance*. 2012;14(1):60. doi:10.1186/1532-429X-14-63

73. Kellman P, Wilson JR, Xue H, et al. Extracellular volume fraction mapping in the myocardium, part 2: initial clinical experience. *Journal of Cardiovascular Magnetic Resonance*. 2012;14(1):61. doi:10.1186/1532-429X-14-64

74. Montone RA, Jang IK, Beltrame JF, et al. The evolving role of cardiac imaging in patients with myocardial infarction and non-obstructive coronary arteries. *Prog Cardiovasc Dis*. 2021;68:78-87. doi:10.1016/j.pcad.2021.08.004

75. Aquaro GD, Ghebru Habtemicael Y, Camastra G, et al. Prognostic Value of Repeating Cardiac Magnetic Resonance in Patients With Acute Myocarditis. *Journal of the American College of Cardiology*. 2019;74(20):2439-2448. doi:10.1016/j.jacc.2019.08.1061

76. Merlo M, Gagno G, Baritussio A, et al. Clinical application of CMR in cardiomyopathies: evolving concepts and techniques: A position paper of myocardial and pericardial diseases and cardiac magnetic resonance working groups of Italian society of cardiology. *Heart Fail Rev*. 2022;28(1):77-95. doi:10.1007/s10741-022-10235-9

77. Rybicki FJ, Udelson JE, Peacock WF, et al. 2015 ACR/ACC/AHA/AATS/ACEP/ASNC/NASCI/SAEM/SCCT/SCMR/SCPC/SNMMI/STR/STS Appropriate Utilization of Cardiovascular Imaging in Emergency Department Patients With Chest Pain. *Journal of the American College of Radiology*. 2016;13(2):e1-e29. doi:10.1016/j.jacr.2015.07.007

78. Grandhi GR, Batlle JC, Maroules CD, et al. Combined stress myocardial CT perfusion and coronary CT angiography as a feasible strategy among patients presenting with acute chest pain to the emergency department. *Journal of Cardiovascular Computed Tomography*. 2021;15(2):129-136. doi:10.1016/j.jcct.2020.06.195

79. Knuuti J, Ballo H, Juarez-Orozco LE, et al. The performance of non-invasive tests to rule-in and rule-out significant coronary artery stenosis in patients with stable angina: a meta-analysis focused on post-test disease probability. *European Heart Journal*. 2018;39(35):3322-3330. doi:10.1093/eurheartj/ehy267

80. Sicari R, Nihoyannopoulos P, Evangelista A, et al. Stress Echocardiography Expert Consensus Statement--Executive Summary: European Association of Echocardiography (EAE) (a registered branch of the ESC). *European Heart Journal*. 2008;30(3):278-289. doi:10.1093/eurheartj/ehn492

81. Pontone G, Guaricci AI, Palmer SC, et al. Diagnostic performance of non-invasive imaging for stable coronary artery disease: A meta-analysis. *International Journal of Cardiology*. 2020;300:276-281. doi:10.1016/j.ijcard.2019.10.046

82. Raff GL, Hoffmann U, Udelson JE. Trials of Imaging Use in the Emergency Department for Acute Chest Pain. *JACC: Cardiovascular Imaging*. 2017;10(3):338-349. doi:10.1016/j.jcmg.2016.10.015

83. Maddahi J, Packard RRS. Cardiac PET Perfusion Tracers: Current Status and Future Directions. *Seminars in Nuclear Medicine*. 2014;44(5):333-343. doi:10.1053/j.semnuclmed.2014.06.011

84. Cavalier JS, Klem I. Using Cardiac Magnetic Resonance Imaging to Evaluate Patients with Chest Pain in the Emergency Department. *J Cardiovasc Imaging*. 2021;29(2):91. doi:10.4250/jcvi.2021.0036

85. Ricci F, Khanji MY, Bisaccia G, et al. Diagnostic and Prognostic Value of Stress Cardiovascular Magnetic Resonance Imaging in Patients With Known or Suspected Coronary Artery Disease: A Systematic Review and Meta-analysis. *JAMA Cardiol*. 2023;8(7):662. doi:10.1001/jamacardio.2023.1290

86. Gulati M, Levy PD, Mukherjee D, et al. 2021 AHA/ACC/ASE/CHEST/SAEM/SCCT/SCMR Guideline for the Evaluation and Diagnosis of Chest Pain: A Report of the American College of Cardiology/American Heart Association Joint Committee on Clinical Practice Guidelines. *Circulation*. 2021;144(22). doi:10.1161/CIR.0000000000001029

87. Vardhanabhuti V, Nicol E, Morgan-Hughes G, et al. Recommendations for accurate CT diagnosis of suspected acute aortic syndrome (AAS)—on behalf of the British Society of Cardiovascular Imaging (BSCI)/British Society of Cardiovascular CT (BSCCT). *BJR*. 2016;89(1061):20150705. doi:10.1259/bjr.20150705

88. Raskob GE, Angchaisuksiri P, Blanco AN, et al. Thrombosis: A Major Contributor to Global Disease Burden. *ATVB*. 2014;34(11):2363-2371. doi:10.1161/ATVBAHA.114.304488

89. Gatti M, Liguori C, Muscogiuri G, et al. Challenges and opportunities to delivering cardiac imaging training: a national survey by the Italian college of cardiac radiology. *Insights Imaging*. 2021;12(1):136. doi:10.1186/s13244-021-01076-5
