# Supplementary material for: SIRM/SIC consensus document on the management of patients with acute chest pain
Source: Radiol Med. 2025 Sep 23;130(12):1936–48. doi: 10.1007/s11547-025-02076-x (PMC12669346; doi:10.1007/s11547-025-02076-x)
Supplement: Supplementary file 2 — Supplementary file2 (DOCX 27 KB) [file 11547_2025_2076_MOESM2_ESM.docx]

**Table S1. Risk Score in patients with Acute Chest Pain**

| Risk Score | Year* | Type | Target Population | Patients | Features | Score range | Outcome |
| --- | --- | --- | --- | --- | --- | --- | --- |
| Chest Pain Score (CPS)^25^ | 2002° | Prospective single-center study in Italy | ACP patients with suspected ACS (≥18 years) | 13762 | ACP Characteristics:  Location (Substernal, precordial [+3], Left chest, neck, lower jaw, epigastrium [+1], Apex [-1])  Radiation (either arm, shoulder, back, neck, lower jaw) [+1]  Character (Crushing, pressing, heaviness [+3]; Sticking, pleuritic, pinprick [-1])  Associated symptoms (Dyspnoea, Nausea, Diaphoresis [+2]; History of angina [+3]) | Range -2 – +12  Very low probability of CAD <4  Low-intermediate  and high probability of CAD ≥4 | In-hospital mortality of overall population / 6-month cardiovascular events (CAD diagnosis; UA; MI; death) of patients at low-risk who underwent evaluation  at ED or CPU |
| EDACS^26^ | 2014 | Prospective study at 2 ED centers in Australia and New Zealand | ACP patients (≥18 years) with suspected ACS | 1974 (derivation cohort) and 608 (validation cohorts) | Age  Sex  Known coronary artery disease or ≥3 risk factors (only applied to patients of 18-50 years)  Diaphoresis  Pain radiates to arm, shoulder, neck, or jaw  Pain occurred or worsened with inspiration  Pain is reproduced by palpation | Range -8 – +34  Low-risk <16 AND • ECG shows no new ischemia AND • 0-hour and 2-hour troponin both negative.  Non-low risk ≥16 or  ECG shows new ischemia or  0-hour or 2-hour troponin positive | 30-day MACE (MI, emergency revascularization, cardiovascular death, ventricular arrhythmia, cardiac arrest, cardiogenic shock, high atrio-ventricular block) |
| HEART^27,28^ | 2008 (derivation study) – 2013 (validation study) | Retrospective study (derivation cohort) at 1 ED center in The Netherlands, and prospective multicenter study (validation cohort) at 10 ED centers in The Netherlands | ACP patients with suspected ACS | 120 (derivation cohort) and 2388 (validation cohort) | History  ECG  Age  Risk factors  Initial sensitive troponin I | Range 0 – 10  Low-risk 0- 3  Intermediate 4-6  High-risk 7-10 | 6-week MACE (MI, PCI, CABG, death) |
| GRACE 1.0^29-31^ | 2003 (first description) – 2004 – 2006 | Registry from 94 hospitals in 14 countries in North and South America, Europe, Australia and New Zealand [7] | UA/NSTE-ACS and STE-ACS | 21688 (derivation cohort) and 22122 (validation cohort) [7] | Age  Heart rate  Systolic blood pressure  Serum creatinine level  CHF (Killip class)  Cardiac arrest at admission  ST-segment deviation (only for in-hospital death)  Elevated cardiac biomarkers of necrosis  In-hospital PCI (only for at discharge to 6-month death/MI)  In-hospital CABG (only for at discharge to 6-month death/MI)  Past history of MI (only for at discharge to 6-month death/MI)  ST-segment depression (only for at discharge to 6-month death/MI) | In-hospital Mortality:  Low risk <109 in NSTE-ACS;  <126 in STE-ACS  Intermediate risk 109–140 in NSTE-ACS; 126–154 in STE-ACS  High risk >140 in NSTE-ACS; >154 in STE-ACS | At admission in-hospital/to 6-month all-cause mortality (or death/MI)  At discharge to 6 months all-cause mortality (or death/MI) |
| GRACE 2.0^32^ | 2014 | Registry from 94 hospitals in 14 countries in North and South America, Europe, Australia and New Zealand | UA/NSTE-ACS and STE-ACS | 32037 (derivation cohort) and 2959 (validation cohort) | Age  Heart rate  Systolic blood pressure  CHF (Killip class)  Serum creatinine level  (if Killip class or creatinine levels are not available, diuretic usage and renal failure can be substituted)  ST-segment deviation  Cardiac arrest at admission  Elevated cardiac enzymes/markers | Range 0 - ≥ 285 | 1-year and 3-year all-cause mortality (or death/MI at 1-year) |
| GRACE 3.0^33^ | 2022 | Consecutive patients of two nation-wide large-scale  registries from UK and Switzerland for model development and external  validation, respectively | NSTE-ACS | 386591 (derivation cohort) and 20727 (external validation cohort) | Age  Heart rate  Systolic blood pressure  Serum creatinine level  Sex  Cardiac arrest at admission  ST-segment deviation  Troponin elevation  Killip class | Score derived by applying a supervised tree-based machine learning algorithm | Sex-specific in-hospital mortality in women and men |
| TIMI^34^ | 2000 | Randomized multicenter controlled trial (unfractionated heparin vs. enoxaparin) at 176 centers in 10 countries in the United States, Canada, South America, and Europe (ESSENCE trial) and at 200 centers across 10 countries in North America, South America, and Europe (TIMI 11B trial) | UA/NSTE-ACS | 1957 (unfractionated heparin, derivation cohort) vs 7081(from three validation cohorts) | Age ≥65 years  ≥3 risk factors for CAD  Use of ASA (last 7 days)  Known CAD (stenosis ≥50%)  >1 episode rest angina in <24 h  ST-segment deviation ≥0.5mm  Elevated cardiac markers | Range 0 – 7  Low-risk 0- 2  Intermediate 3-4  High-risk 5-7 | All-cause mortality, new or recurrent MI, or severe recurrent ischemia requiring urgent revascularization through 14 days |
| TIMI^35^ | 2000 | Large multicenter trial (InTIME II trial) at >800 centers worldwide for the derivation cohort; randomized multicenter controlled trial (TIMI 9) for the external validation cohort | STE-ACS | 14114 (derivation cohort) and 3687 (external validation cohort) | Age  Systolic blood pressure) <100 mmHg  Heart rate >100 per min  Killip class II-IV  STEMI of anterior wall of heart or left bundle branch block in ECG  Diabetes or history of hypertension or angina  Weight of patient < 67 kg  Time to treatment > 4h | Range 0 – 14 | 30-day mortality |
| PURSUIT^36^ | 2000 | Randomized controlled trial (eptifibatide vs. placebo) at 726 centers in 28 countries in western and eastern Europe and North and South America | UA/NSTE-ACS | 9461 | Age in decade  Sex  Worst CCS-class in previous 6 weeks  Signs of heart failure  ST-depression on presenting ECG | Range 0 – 18 | 30-day mortality (or death/MI) |
| Killip Class^37-39^ | 1967^§^ -2003 - 2019 | Multi-center study from 4 large clinical trials (GUSTO IIb, PARAGON A, PARAGON B, and PURSUIT) [14].  National ACS Israeli survey [15] | NSTE-ACS and STE-ACS | 26090 NSTE-ACS patients [14]  9736 ACS patients (enrolled in the years 2000 to 2008) and 5288 ACS patients (enrolled in the time-period 2010 to  2016) [15] | Quantifies severity of heart failure in ACS:  Class I – No signs of congestion  Class II – S3 gallop, basal rales on auscultation, and/or jugular vein distension (findings consistent with mild to moderate heart failure)  Class III – Acute pulmonary edema  Class IV – Cardiogenic shock | Killip classes I-IV | 30-day mortality [14]  30-day and 1-year mortality, and 30-day MACE (death, MI, stroke, UA, stent thrombosis, urgent revascularization) [15] |
| Wells’ Criteria^40^ | 2001 | Prospective multicenter study at 4 ED  centers in Canada | Patients ≥18 years with suspected PE | 930 | Clinical signs and symptoms of DVT  Pulmonary embolism as  likely as or more likely than an alternative diagnosis  Heart rate >100  Immobilization at least 3 days or surgery in the previous 4 weeks  Previous, objectively diagnosed PE or DVT  Hemoptysis  Malignancy (patients who were receiving cancer treatment, those in whom treatment had been stopped within the past 6 months, or those who were receiving palliative care) | Range 0 – 12.5  Low probability of PE <2  Moderate probability of PE 2-6  High probability of PE >6  “PE Unlikely” ≤ 4  “PE Likely” >4 | Occurrence of a thromboembolic event during 3-month follow-up |
| PERC^41^ | 2004 | Prospective multicenter study at 10 ED centers in the United States | Suspected PE in patients with a low clinical probability of PE | 3148 (derivation cohort) and 1427 (low-risk validation cohort) and 382 (very low-risk validation cohort) | Age ≥50  Heart rate ≥100  Oxygen saturation on room air <95%  Unilateral leg swelling  Hemoptysis  Recent trauma or surgery  Prior PE or DVT  Hormone use (oral contraceptives, hormone replacement or estrogenic hormones use in males or female patients) | Range 0 – 8  A PERC score of 0 (PERC-negative) suggests a low risk of PE.  A PERC evaluation is considered positive if any one of the eight criteria are met | Occurrence of a thromboembolic event during 3-month follow-up |
| Revised Geneva^42^ | 2006 | Prospective multicenter study at two ED centers in Switzerland and one center in France | Suspected PE | 956 (derivation cohort) and 749 (validation cohort) | Age >65  Previous DVT or PE  Surgery (under general anesthesia) or lower limb fracture in past month  Active malignant condition (solid or hematologic malignant condition, currently active or considered cured <1 year)  Unilateral lower limb pain  Hemoptysis  Heart rate  Pain on lower-limb deep venous  palpation and unilateral edema | Range 0 – 22  Low risk 0-3  Intermediate risk 4-10  High-risk ≥11 | Occurrence of a thromboembolic event during 3-month follow-up |
| PESI^43^ | 2005 | Large statewide Pennsylvania database for derivation and internal validation; prospective external validation at two centers in Switzerland and France | Patients ≥18 years diagnosed with PE | 10354 (derivation cohort), 5177 (internal validation cohort), and 221 (external validation cohort) | Age, per year  Sex  History of cancer  History of heart failure  History of chronic lung disease  Heart rate ≥110  Systolic BP <100 mmHg  Respiratory rate ≥30  Temperature <36°C/96.8°F  Altered mental status (disorientation, lethargy, stupor, or coma)  Arterial oxygen saturation <90% | Very low risk ≤ 65  Low risk 66-85  Intermediate risk 86-105  High-risk 106-125  Very high risk >125 | 30-day mortality |
| ADD-RS^44^ | 2011 | Retrospective validation in patients with known AD from the IRAD database (24 centers) | Suspected AD | 2538 | Any high-risk condition (Marfan syndrome or other connective tissue disease, family history of aortic disease, known aortic valve disease, recent aortic manipulation, or known TAA)  Any high-risk pain feature (Chest, back, or abdominal pain described as abrupt onset, severe intensity, or ripping/tearing)  Any high-risk exam feature (Evidence of perfusion deficit [pulse deficit, systolic BP differential, or focal neurological deficit plus pain], new aortic insufficiency murmur [with pain], hypotension/shock) | Range 0 – 3  Low risk 0  Intermediate risk 1  High risk 2-3  Sensitivity for AD of 95.7% for ADD-RS >1 | Sensitivity in predicting AD |
| ADD-RS / D-Dimer^45^ | 2018 | Prospective multicenter study (ADvISED study) at 6 ED centers in 4 countries (Brazil, Germany, Italy, Switzerland) | Patients >18 years with suspected AAS | 1850 | ADD-RS (12 variables in 3 categories) plus D-dimer (negative if <500 ng/mL fibrinogen equivalent units) | Range ADD-RS: 0 – 3  D-dimer: positive/negative.  ADD-RS ≤1/D-dimer negative: non–high-risk  ADD-RS score > 1 should proceed to CTA or other conclusive imaging irrespective of D-dimer levels | Failure rate of 2 diagnostic strategies ruling out AAS (ADD-RS=0/D-dimer negative and ADD-RS ≤1/D-dimer negative).  Final case adjudication of AAS was based on conclusive diagnostic imaging, autopsy, surgery, or 14-day follow-up |

*: first year of Risk Score description, unless otherwise specified. °A scoring system for angina pectoris symptoms was historically described in 1968 by Frank CW [Frank CW. The course of coronary heart disease: factors relating to prognosis. Bull N Y Acad Med. 1968 Aug;44(8):900-15]. ^§^Developed by Killip and Kimball in 1967 before reperfusion therapy (PCI, thrombolytics); **AAS**, acute aortic syndrome; **ACP**, acute chest pain; **ACS**, acute coronary syndrome; **AD**, aortic dissection; **ADD-RS**, Aortic Dissection Detection Risk Score; **ADvISED,** Aortic Dissection Detection Risk Score Plus D-Dimer in Suspected Acute Aortic Dissection; **CAD**, coronary artery disease; **CABG**, coronary artery bypass graft; **CCS**, Canadian Cardiovascular Society; **CPU**, Chest pain unit; **CTA**, computed tomography angiography; **DVT**, deep venous thrombosis; **ECG,** electrocardiography; **ECG**, electrocardiogram; **ED**, emergency department; **EDACS,** ED Assessment of Chest Pain Score; **ESSENCE**, Efficacy and Safety of Subcutaneous Enoxaparin in Unstable Angina and Non-Q-Wave MI; **GRACE**, Global Registry of Acute Coronary Events; **HEART**, History, Electrocardiography, Age, Risk factors, and Troponin; **InTIME II**, Intravenous nPA for Treatment of Infarcting Myocardium Early II; **IRAD**, International Registry of Acute Aortic Dissection; **MACE,** Major Adverse Cardiac Event; **MI**, myocardial infarction; **NSTE-ACS**, non-ST elevation acute coronary syndrome; **PCI**, percutaneous coronary intervention; **PE**, pulmonary embolism; **PESI**, Pulmonary Embolism Severity Index; **PTCA**, percutaneous transluminal coronary angioplasty; **PURSUIT**, Platelet glycoprotein IIb/IIIa in Unstable angina: Receptor Suppression Using Integrilin (eptifibatide) Therapy; **STE-ACS,** ST elevation acute coronary syndrome; **STEMI**, ST-segment elevation myocardial infarction; **TAA**, thoracic aortic aneurysm; **TIMI**, Thrombolysis In Myocardial Infarction; **UA**, unstable angina.
